# An Environment-Tolerant Ion-Conducting Double-Network Composite Hydrogel for High-Performance Flexible Electronic Devices

**DOI:** 10.1007/s40820-023-01311-2

**Published:** 2024-01-29

**Authors:** Wenchao Zhao, Haifeng Zhou, Wenkang Li, Manlin Chen, Min Zhou, Long Zhao

**Affiliations:** 1 State Key Laboratory of Advanced Electromagnetic Technology, School of Electrical and Electronic Engineering, Huazhong University of Science and Technology, Wuhan, 430074 People’s Republic of China; 2 School of Chemistry and Chemical Engineering, Huazhong University of Science and Technology, Wuhan, 430074 People’s Republic of China

**Keywords:** Ionic liquids, Double-network hydrogels, Temperature tolerance, Multifunctionality, Flexible electronic devices

## Abstract

High-performance ion-conducting hydrogels (ICHs) are vital for developing flexible electronic devices. However, the robustness and ion-conducting behavior of ICHs deteriorate at extreme temperatures, hampering their use in soft electronics. To resolve these issues, a method involving freeze–thawing and ionizing radiation technology is reported herein for synthesizing a novel double-network (DN) ICH based on a poly(ionic liquid)/MXene/poly(vinyl alcohol) (PMP DN ICH) system. The well-designed ICH exhibits outstanding ionic conductivity (63.89 mS cm^−1^ at 25 °C), excellent temperature resistance (− 60–80 °C), prolonged stability (30 d at ambient temperature), high oxidation resistance, remarkable antibacterial activity, decent mechanical performance, and adhesion. Additionally, the ICH performs effectively in a flexible wireless strain sensor, thermal sensor, all-solid-state supercapacitor, and single-electrode triboelectric nanogenerator, thereby highlighting its viability in constructing soft electronic devices. The highly integrated gel structure endows these flexible electronic devices with stable, reliable signal output performance. In particular, the all-solid-state supercapacitor containing the PMP DN ICH electrolyte exhibits a high areal specific capacitance of 253.38 mF cm^−2^ (current density, 1 mA cm^−2^) and excellent environmental adaptability. This study paves the way for the design and fabrication of high-performance multifunctional/flexible ICHs for wearable sensing, energy-storage, and energy-harvesting applications.

**Supplementary Information:**

The online version contains supplementary material available at 10.1007/s40820-023-01311-2.

## Highlights

- Novel double-network (DN) ion-conducting hydrogel (ICH) based on a poly(ionic liquid)/MXene/poly(vinyl alcohol) system (named PMP DN ICH) was synthesized using freeze–thawing and ionizing radiation technology.
- The PMP DN ICH possesses a multiple cross-linking mechanism and exhibits outstanding ionic conductivity (63.89 mS cm^−1^), excellent temperature resistance (−60–80 °C) and decent mechanical performance.
- The well-designed PMP DN ICH shows considerable potential in wearable sensing, energy storage, and energy harvesting.

**Supplementary Information:**

The online version contains supplementary material available at 10.1007/s40820-023-01311-2.

## Graphical abstract

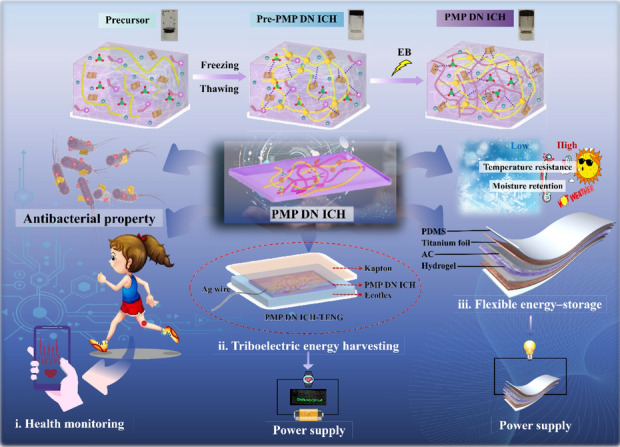

## Introduction

Ion-conducting hydrogels (ICHs) have been extensively used to develop electronic skin (e-skin), wearable sensors, supercapacitors (SCs), and triboelectric nanogenerators (TENGs) owing to their inherent properties such as flexibility, biocompatibility, and high conductivity [1–3]. However, conventional conductive hydrogels contain substantial amounts of absorbed water, which inevitably freezes or evaporates at cold or high temperatures, respectively. The flexibility and conductivity deteriorate significantly, which severely inhibits their performance in practical applications [4, 5]. Additionally, they tend to exhibit inferior mechanical properties [6, 7]. Therefore, designing new ICHs with high environmental tolerance and decent mechanical stability without sacrificing ionic conductivity is challenging but crucial.

Several attempts have been made to address these problems. On the one hand, ICHs with excellent temperature resistance and water retention have been obtained by utilizing organic solvents [8], inorganic salts [9], or ionic liquids (ILs) [10] as cryoprotectants/humectants. Unfortunately, organic solvents such as ethylene glycol and glycerol hinder ion migration, resulting in ICHs with poor ionic conductivity [7, 9]. Alternatively, ICHs containing a large amount of inorganic salts exhibit a salting-out effect, which increases their cross-linking degree while restricting ion migration [11]. ILs which are organic molten salts comprising organic cations and organic/inorganic anions have drawn considerable interest in the development of electronic devices owing to their benefits including high ionic conductivity, high thermal/chemical stability, nonvolatile behavior, and wide electrochemical window [12, 13]. Furthermore, the chemical structure and performance of ILs can be tuned by changing the anion–cation pairs [1, 14]. However, ILs are unable to maintain a stable shape owing to their viscous flow characteristics, and leakage problems occur when they are doped into polymer matrices [15]. Therefore, by polymerizing IL monomers into poly(ionic liquid)s (PILs), the inherent properties of the ILs can be transferred to the polymer chain and the potential leakage problem of the ILs can be eliminated, thereby obtaining ICHs with excellent temperature resistance and ionic conductivity [16, 17].

On the other hand, strategies have been reported for synthesizing diverse strong and tough hydrogels such as double-network (DN) hydrogels [18] and nanocomposite hydrogels [19, 20]. Among these, DN hydrogels can effectively disperse stress and dissipate energy by introducing sacrificial bonds such as hydrogen bonds and ionic bonds into the network, which improve flexibility [18, 21].

**Fig. 1:**
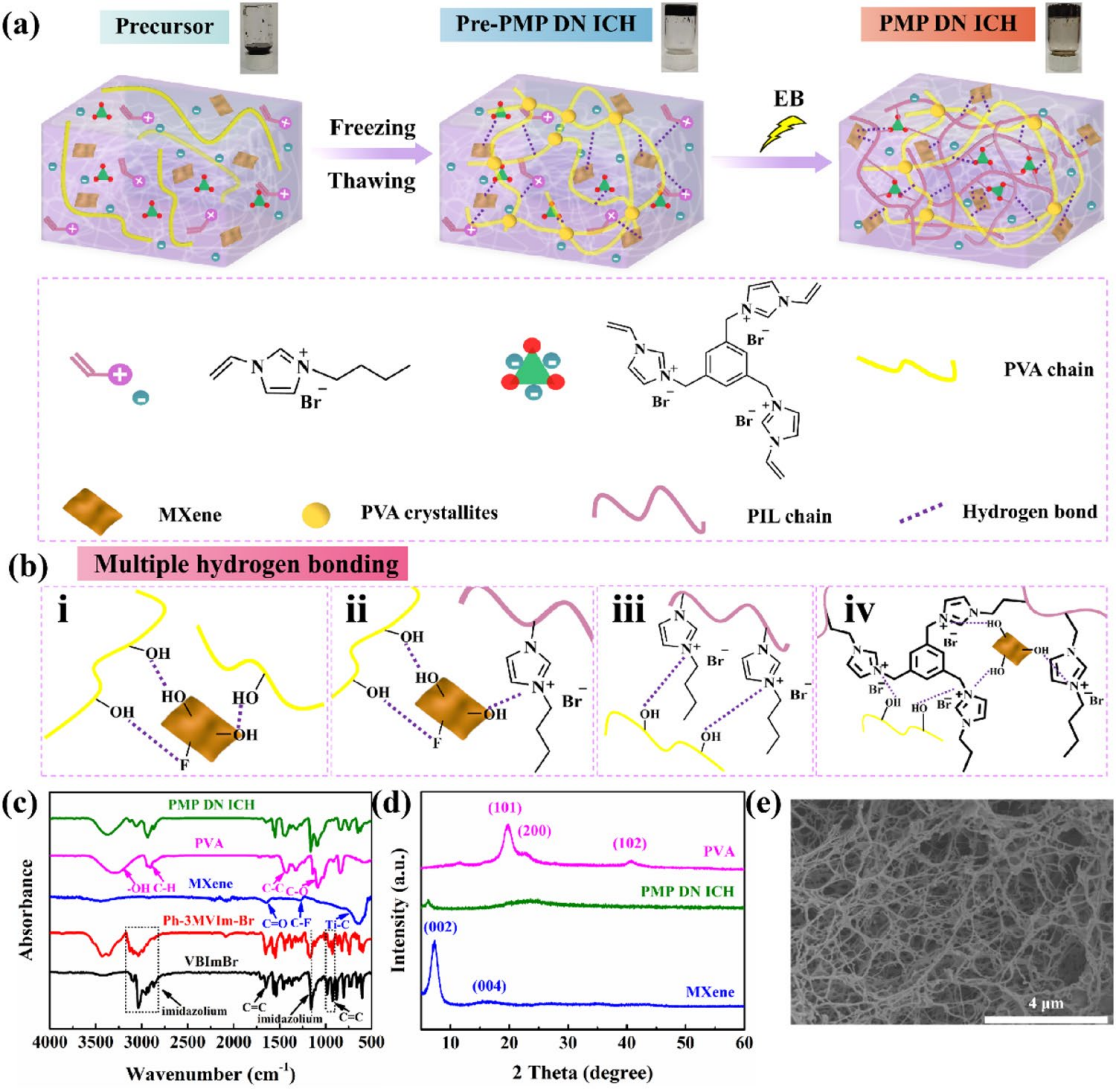
Preparation and characterization of the PIL/MXene/PVA (PMP)-based double-network (DN) ion-conducting hydrogel (ICH). Schematics illustrating the **a** construction of PMP DN ICH and its **b** multiple hydrogen bond interactions. **c**, **d** FTIR and XRD spectra of VBImBr, Ph–3MVIm–Br, the MXene, PVA, and PMP DN ICH. **e** SEM image of PMP DN ICH

Poly(vinyl alcohol (PVA) has been widely used as a hydrogel polymer matrix because of its excellent biocompatibility, water-retaining ability, nontoxicity, and low cost [22, 23]. Several PVA-based DN hydrogels with excellent mechanical properties and freezing resistance have recently been reported [24, 25]. However, these hydrogels must exhibit sufficient conductivity to be applicable as flexible electronic materials. Therefore, a novel strategy was devised in the present study to construct a multifunctional PIL/MXene/PVA (PMP)-based DN ICH (denoted as PMP DN ICH) with excellent temperature tolerance, mechanical flexibility, and superior ionic conductivity by combining freeze–thawing and ionizing radiation methods (Fig. 1a). The freeze-thawing process enabled in situ crystallization of PVA, which facilitated the formation of a physically cross-linked network [26], whereas the subsequent ionizing radiation process allowed the formation of a chemically cross-linked PIL–PVA network. The large amount of the PIL and its network helped eliminate the potential leakage problem of ILs and improve the temperature resistance of the PVA-based hydrogel while endowing it with excellent ionic conductivity. Moreover, nanosheets of a two-dimensional (2D) transition metal carbide/nitride (Ti_3_C_2_T_x_ MXene) with abundant surface hydrophilic groups (such as –F, –OH, and =O) were introduced as physical cross-linkers to further enhance the mechanical properties of PMP DN ICH by forming noncovalent interactions, such as hydrogen bonding, with the polymer network [27].

Finally, the DN structure formed through a multiple cross-linking mechanism enabled PMP DN ICH to exhibit outstanding ionic conductivity, wide-ranging temperature resistance, and decent mechanical properties. More importantly, PMP DN ICH was used to construct a flexible strain sensor, a thermal sensor, an all-solid-state SC, and a single-electrode TENG that exhibited reliable properties. Overall, the comprehensive performance of PMP DN ICH makes it a versatile candidate material for fabricating flexible electronic devices intended for wearable sensing, energy-storage, and energy-harvesting applications.

## Experimental Section

### Materials

1-Vinyl-3-butylimidazolium bromide (VBImBr, 99%) was purchased from the Lanzhou Institute of Chemical Physics (China). 1-vinylimidazole (≥ 99%), 2,6-ditert-butyl-4-methylphenol (BHT, 99%), and 1,3,5-tris(bromomethyl)benzene (98%) were purchased from Sigma-Aldrich. PVA was purchased from Sinopharm Chemical Reagent Co., Ltd. (China), with an average degree of polymerization (DP) of 1750 ± 50. Activated carbon (YP–50F) and carbon black were purchased from Kuraray Co., Ltd and 9 Ding Chemistry Co., Ltd, respectively. Ecoflex 00–50 silicone rubber was purchased from Smooth-On Company, USA. All other chemicals (analytical grade) were purchased from Sinopharm Chemical Reagent Co., Ltd. (China), and used without further purification.

### Preparation of Ti_3_C_2_T_x_ MXene Nanosheets

Ti_3_C_2_T_x_ MXene (MXene) was synthesized according to the following process. First, 1 g LiF was added to 40 mL HCl solution (9 mol L^−1^) and stirred for 30 min. Secondly, 1 g Ti_3_AlC_2_ powder was slowly added to the above solution and stirred at 35 °C for 48 h. Finally, the reaction solution was washed by centrifugation with water until the pH of the solution was ≥ 6, and the precipitate was collected. Subsequently, the precipitate was sonicated with ethanol and water, and was centrifuged at 3500 r min^−1^ for 25 min; the upper dark green liquid was the delaminated MXene nanosheet aqueous solution.

### Synthesis of Ph–3MVIm–Br

1,3,5-tris (1′-methylene-3′-vinylimidazolium bromide) benzene (termed Ph–3MVIm–Br; Ph = phenyl, 3 denotes the number of branched polymerizable ion pairs, MVIm = 1-methylene-3-vinylimidazolium cation, Br = bromide anion) was synthesized according to Ref. [28]; the detailed synthesis process was shown in the supporting information.

### Radiation Synthesis of PMP DN ICH

Firstly, 1 g PVA power was added to 9 g deionized water under stirring at 90 °C for 4 h until PVA was completely dissolved to form 10 wt% PVA solution. Then, VBImBr (1.848 g, 8 mol L^−1^), Ph–3MVIm–Br (12.77 mg, 0.02 mol L^−1^), MXene (27.72 mg, 1.5 wt%, relative to monomer), and 10% PVA aqueous solution (1 mL) were added into an irradiation flask and ultrasonic dispersed. Subsequently, the solution was bubbled by N_2_ for 10 min. The above solution was placed at − 25 °C for 12 h and then thawing 6 h to forming physical cross-linked gel. Finally, the above physical cross-linked gel was irradiated under an electron-beam (EB) accelerator (10 MeV, EL PONT Co., Ltd. China) with the dose rate of 10 kGy pass^−1^ to obtain PMP DN ICH.

### General Characterization Techniques

The chemical structure of the Ph–3MVIm–Br was analyzed by ^1^H nuclear magnetic resonance (^1^H NMR) using D_2_O as the solvent. Fourier-transform infrared (FTIR, Tensor 27, Bruker) spectrometry and scanning electron microscope (SEM, SU8000, Hitachi, Japan) were used to confirm the structure and composition of the freeze-dried PMP DN ICH. The thermal stability of the PMP DN ICH was carried on a thermogravimetric analysis (TGA) (Q600 SDT) under N_2_ gas atmosphere with a heating rate of 10 °C min^−1^. Differential scanning calorimetry (DSC) was performed using a TA Q200 instrument. X-ray diffraction (XRD) was tested by Rigaku corporation (Japan) diffractometer. Cyclic voltammetry (CV), electrochemical impedance spectroscopy (EIS), galvanostatic charge/discharge (GCD) were measured by an electrochemical workstation (CHI660E, Chenhua, Shanghai). The cycling stability was tested with a LANBTS electrochemical instrument. The open-circuit voltage, short-circuit current, and transferred charge amount were recorded by a Keithley 6514 electrometer.

### Conductivity Tests

The ionic conductivity of the PMP DN ICH was measured on the electrochemical workstation. The PMP DN ICH (diameter of 13 mm, thickness of approximately 8 mm) was sandwiched between two sheet metal electrodes. Next the electrochemical impedance spectroscopy (EIS) was measured at different temperatures (− 60, − 25, 0, 25, 40, 60, and 80 °C). It was calculated by Eq. (1):

1 $$ \sigma = l/(R \times S) $$

where *l* (mm) represents the thickness of the PMP DN ICH, *R* (Ω) represents the bulk resistance, and *S* (mm^2^) represents the contact area of the PMP DN ICH. The samples were tested in parallel in three groups.

### Measurement of Adhesive Strength and Mechanical Properties

The adhesive strength and mechanical properties of the PMP DN ICH were measured by using the tensile testing machine (CMT–4104, Shenzhen sans testing machine Co., Ltd.). The adhesive strength testing method of the PMP DN ICH is shown in Fig. S12c. Cut the PMP DN ICH into regular-shaped samples (30 mm × 20 mm × 3 mm), and sandwich them between two substrates. Next, the tensile testing machine with a 100 N load cell broken the PMP DN ICH at a rate of 50 mm min^−1^. The experimental results of the adhesive strength were based on the results of at least 3 samples. Besides, the cyclic tensile tests of the PMP DN ICH (40 mm × 10 mm × 3 mm) were also carried out on the tensile testing machine under the rate of 200 mm min^−1^.

### Electrical Sensing Properties of the PMP DN ICH-Based Strain Sensor

The sensing properties of the PMP DN ICH-based strain sensor were evaluated by the relative changes of the resistance. By loading the PMP DN ICH onto the tensile testing machine and connecting it to the electrochemical workstation with copper wires, the strain-induced resistance change of the PMP DN ICH can be monitored in real time. The relative rate of change of the resistance was calculated by Eq. (2):

2 $$ \Delta R/R0 = (R - R0)/R0 \times 100\% $$

where *R* and *R*_0_ are the resistance without and with stretching, respectively.

### Human Motion Detection of the PMP DN ICH-Based Strain Sensor

The PMP DN ICH was cut into a cuboid shape (40 mm × 10 mm × 3 mm) and adhered to the various joints of the human body (finger, wrist, neck, belly, and knee) to detect the human motion. Then, the two ends of the PMP DN ICH were connected to an electrochemical workstation or a small portable wireless transmission device. This experiment was completed with the assistance of a volunteer, and informed written consent was obtained for publishing the images and data. The gel was not harmful to humans.

### Thermal Detection

The resistance of the sensor at various temperatures was measured by the electrochemical workstation. The temperature coefficient of resistance (TCR) was calculated via Eq. 3:

3 $$ {\text{TCR}} = (1/R0) \times (\Delta R/\Delta T) $$

where *R*_0_ (*Ω*): the initial resistance of the PMP DN ICH at 25 °C; Δ*R* (*Ω*): the relative change of resistance; and Δ*T* (°C): the relative change of temperature.

### Fabrication and Electrochemical Measurement of the Flexible All-Solid-State Supercapacitor

An appropriate amount of NMP solution was added dropwise to the mixture of activated carbon, carbon black, and PVDF powder with a mass ratio of 85:5:10. After fully grinding and uniform, the mixed slurry was applied to the cleaned rectangle nickel foam (1 × 2 cm^2^) and dried in vacuum at 50 °C for 48 h. The total mass load of electrode material was about 3.0 mg. Then, the activated carbon electrode was performed by a powder tablet press (YP–40 T, Tianjin Jinfulun technology Co., Ltd) under a pressure of 10 MPa. The SCs was assembled by the activated carbon electrodes and the PMP DN ICH with a sandwich structure. Finally, two titanium foils as current collector and conductive wire were placed on both sides of the sample and sealed with PDMS. Electrochemical performance was tested after standing 1 h.

### Fabrication of the Single-Electrode PMP DN ICH–TENG

The PMP DN ICH–TENG was fabricated by sandwiching the PMP DN ICH with commercial kapton film and ecoflex, where PMP DN ICH, kapton film, and ecoflex classed as the electrode, the negative and positive electrification layer, respectively. And the Ag wire was attached to PMP DN ICH for electrical output measurements.

## Results and Discussion

### Design and Preparation of PMP DN ICH

MXene nanosheets were synthesized according to our previously reported method [29]. The IL 1,3,5-tris(1′-methylene-3′-vinylimidazolium bromide) benzene (Ph–3MVIm–Br) was used as a cross-linker after being synthesized by alkylation. The chemical structure of Ph–3MVIm–Br was determined by ^1^H nuclear magnetic resonance spectroscopy (Fig. S1). PMP DN ICH was prepared using a two-step method (Fig. 1a) that involved dissolving the IL monomer 1-vinyl-3-butylimidazolium bromide (VBImBr) and IL cross-linker Ph–3MVIm–Br in a 10% aqueous PVA solution, and then ultrasonically dispersing a certain amount of the MXene into the preceding solution. A physically cross-linked network was readily obtained from the resulting black precursor solution by freeze–thawing. Subsequently, a chemically cross-linked PIL–PVA network was constructed by the in situ polymerization/cross-linking driven by the ionizing radiation technique. Finally, black-colored PMP DN ICH with abundant covalent and noncovalent cross-linked networks was successfully obtained (Fig. 1b). The radiation-associated conditions for synthesizing PMP DN ICH were comprehensively optimized (Figs. S2 and S3) and determined to be as follows: absorbed dose, 20 kGy; monomer concentration, 8 mol L^−1^; cross-linker concentration, 0.02 mol L^−1^; and MXene content, 1.5 wt%. The gel synthesized under these conditions was used in the subsequent experiments.

Fourier-transform infrared (FTIR) spectra of VBImBr, Ph–3MVIm–Br, the MXene, PVA, and PMP DN ICH were acquired (Fig. 1c). The imidazolium-associated peaks at 1157 and ~ 3000 cm^−1^ appeared in the spectrum of PMP DN ICH after the irradiation step, indicating that the imidazole ring structure was not damaged by irradiation. Moreover, the characteristic –C=C– peaks at 924–981 and 1648 cm^−1^ almost disappeared after the irradiation, indicating successful polymerization/cross-linking of PMP DN ICH [30]. Additionally, peaks appeared at 3296 and 1086 cm^−1^, presumably owing to the stretching vibrations of –OH groups in the PVA chains [31]; these peaks shifted to 3367 and 1093 cm^−1^, respectively, in the spectrum of PMP DN ICH, suggesting hydrogen bond formation [32]. X-ray diffractometry (XRD) analysis of PVA, the MXene, and PMP DN ICH (Fig. 1d) indicated that the typical PVA crystalline peaks corresponding to the (101), (200), and (102) lattice planes almost completely receded in the pattern of PMP DN ICH, suggesting that the PVA in PMP DN ICH could be chemically cross-linked [33]. Additionally, the intensity of the (002) peak of the MXene in PMP DN ICH decreased and shifted to a lower 2*θ* value, implying that the *d*-spacing of the MXene could have increased slightly, possibly owing to hydrogen bonding between the MXene and the other components [34, 35]. Scanning electron microscopy (SEM) revealed the highly porous nature of PMP DN ICH (Fig. 1e), and energy-dispersive X-ray spectroscopy (EDS) mapping confirmed the uniform distribution of Ti in PMP DN ICH, suggesting a regular distribution of the MXene (Fig. S4). Overall, these findings validated the synthesis of PMP DN ICH with a homogeneously cross-linked network featuring abundant covalent and noncovalent interactions using the aforementioned meticulous design approach.

### Mechanical Properties, Environmental Tolerance, and Conductivity of PMP DN ICH

**Fig. 2:**
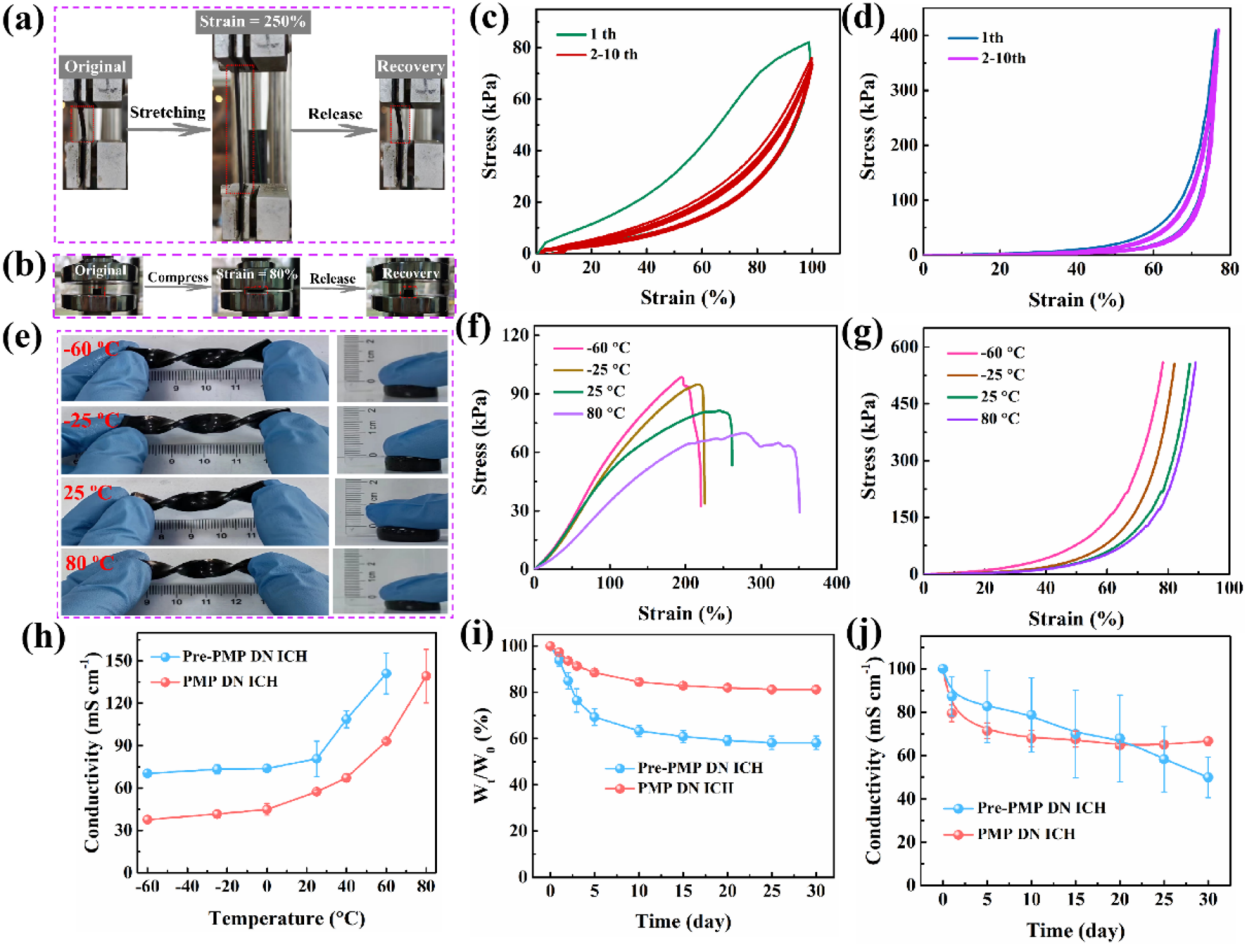
**a**, **b** Photographs of PMP DN ICH undergoing stretching and compression. **c**, **d** Cyclic tensile and compressive loading–unloading curves of PMP DN ICH at strains of up to 100% and 70% for 10 successive cycles. **e** Photographs showing the temperature resistance behavior of PMP DN ICH. **f**, **g** Tensile and compressive stress–strain curves of PMP DN ICH acquired from − 60–80 °C. **h** Conductivities of Pre-PMP DN ICH and PMP DN ICH from − 60–80 °C. **i**, **j** Changes in weight and conductivity of Pre-PMP DN ICH and PMP DN ICH during 30 d of storage in an ambient environment. The error bars represent standard deviation; sample size *n* = 3

The mechanical performance, environmental tolerance, and conductivity of PMP DN ICH were explored to determine its applicability in flexible electronic devices. Moreover, when the PMP DN ICH was attached on the skin surface as a wearable material to detect human movement, adhesion was also key influencing factors. As shown in Fig. S5, PMP DN ICH exhibited enhanced mechanical performance owing to its chemically cross-linked structure that was generated by ionizing radiation. Moreover, it showed remarkable mechanical elasticity and shape recovery, given its ability to undergo large stretching and compression without any evident structural deformation and then quickly return to its initial shape after stress removal (Fig. 2a, b). PMP DN ICH was subjected to 10 successive tensile–relaxation cycles (*ε* = 100%) and compression–relaxation cycles (*ε* = 70%) to quantitatively examine its fatigue resistance and cycling stability (Fig. 2c, d). After displaying hysteresis in the first loading–unloading cycle, the stress–strain curves of PMP DN ICH in the subsequent cycles almost overlapped, indicating decent elastic behavior and fatigue resistance [36, 37]. In contrast, the hydrogel precursor Pre-PMP DN ICH exhibited inferior fatigue resistance and cycling stability (Fig. S6a–d).

To achieve practical viability, ICHs must exhibit acceptable performance under extreme conditions in terms of aspects such as temperature resistance, long-term stability, and anti-drying properties. To that end, the temperature resistance of Pre-PMP DN ICH and PMP DN ICH was analyzed under extreme storage conditions (Figs. 2e and S6e). The results indicated that both materials exhibited outstanding flexibility in terms of being effortlessly twisted/compressed and being bent at − 60 °C. However, Pre-PMP DN ICH which had a physically cross-linked structure could not be used at high temperatures because its structure was destroyed after only 10 min at 80 °C (Fig. S7). Temperature-dependent tensile and compressive stress–strain curves of Pre-PMP DN ICH (Fig. S6f, g) and PMP DN ICH (Fig. 2f, g) suggested that the mechanical strength increased and the ductility decreased with decreasing temperature. Notably, PMP DN ICH exhibited satisfactory tensile stress–strain and compressive stress–strain characteristics at − 60 °C (98.6 kPa–195% and 559.4 kPa–78.3%, respectively) in terms of meeting practical application requirements. This level of mechanical flexibility of PMP DN ICH at extreme temperatures can be leveraged to significantly broaden its applicability.

The conductivities of Pre-PMP and PMP DN ICH at different temperatures were investigated by electrochemical impedance spectroscopy (EIS) (Figs. 2h and S8). The conductivity of Pre-PMP DN ICH increased from 70.26 mS cm^−1^ at − 60 °C to 141.08 mS cm^−1^ at 60 °C, whereas that of PMP DN ICH increased from 37.65 mS cm^−1^ at − 60 °C to 139.21 mS cm^−1^ at 80 °C. The results indicate the stable ion-transporting tendency of the DN ICH at extreme temperatures. The excellent conductivity of PMP DN ICH in harsh environments was corroborated by comparison with those of previously reported temperature-tolerant hydrogels (Fig. S9). Additionally, PMP DN ICH was used as a wire in a circuit to illuminate a light-emitting diode (LED) using a 3 V power source (Fig. S10 and Movies S1, S2). Stretching or compressing PMP DN ICH led to commensurate changes in the brightness of the LED lamp.

The long-term stability and anti-drying property of the DN ICH were also explored (Figs. 2i, j and S11–S13). After storage for 30 d in an ambient environment, Pre-PMP DN ICH showed significant volume shrinkage owing to water evaporation (Fig. S11), with its weight and conductivity decreasing to 58.19% and 49.92%, respectively. In contrast, PMP DN ICH exhibited relatively higher final weight and conductivity values (81.20% and 66.66%, respectively). The excellent long-term stability and moisture retention of PMP DN ICH were evidently due to the abundant hydrogen bonds in the formed three-dimensional network, which effectively reduced the evaporation rate of internal water [38, 39]. Furthermore, the MXene in PMP DN ICH also exhibited excellent oxidation resistance (Fig. S14) [40]. More importantly, PMP DN ICH could be functioned as an effective adhesive material (Fig. S15).

To sum up, the outstanding mechanical performance, environmental resistance, long-term moisture retention ability, conductivity, and oxidation resistance of PMP DN ICH underscore its application potential in flexible wearable electronic device fabrication.

### Antibacterial Performance of PMP DN ICH

**Fig. 3:**
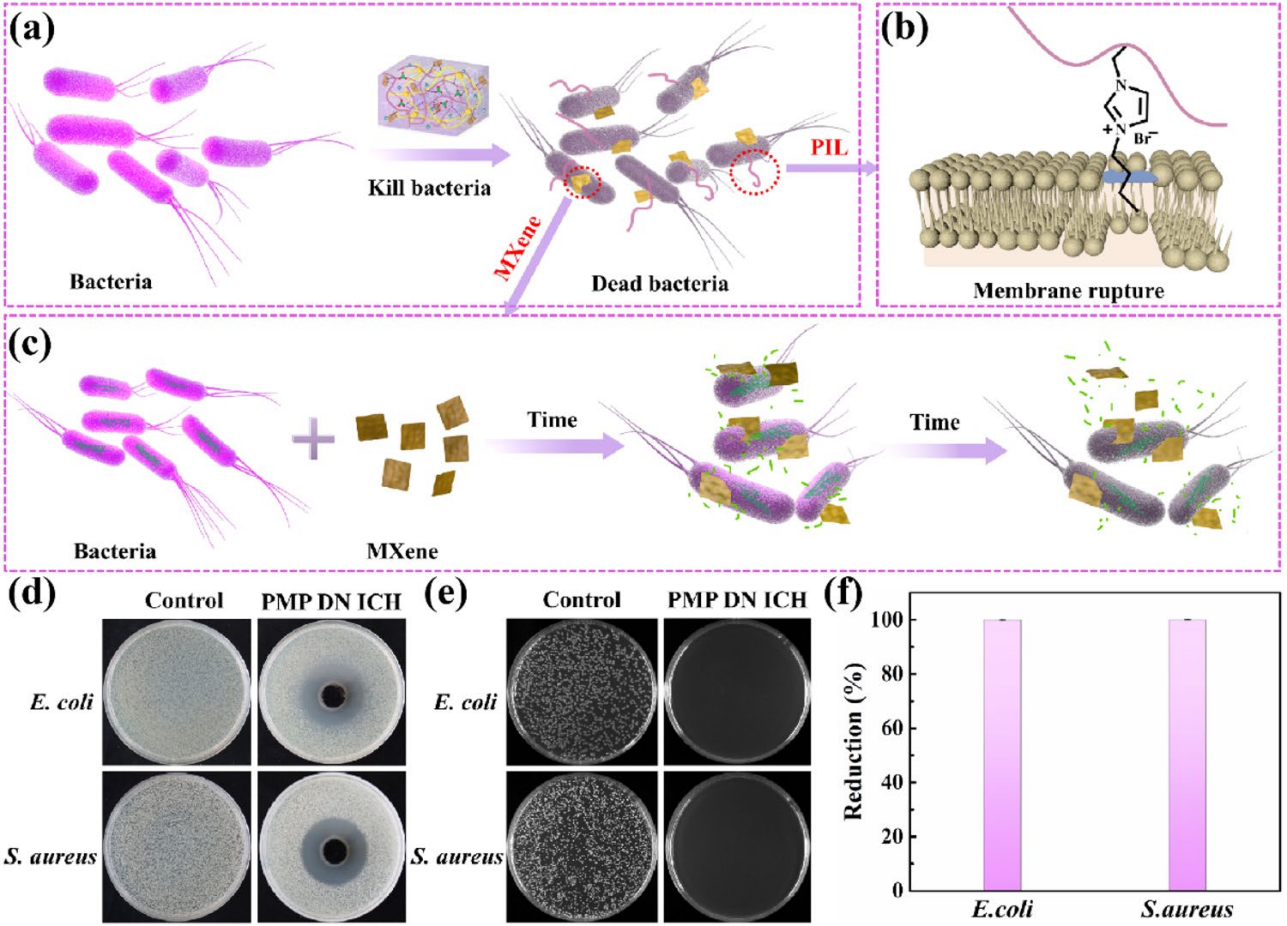
**a**–**c** Antibacterial schematic illustration of the PMP DN ICH. **d**–**f** The antibacterial activity against *E. coli* and *S. aureus* of the PMP DN ICH

Antibacterial performance plays a critical role to wearable material. Many studies have proved that imidazole-based ILs and MXene possessed outstanding antibacterial performance [41, 42]. Therefore, the antibacterial activities of the fabricated PMP DN ICH were verified by using Gram-negative *Escherichia coli* (*E. coli*) and Gram-positive *Staphylococcus aureus* (*S. aureus*). Figure 3a–c depicts the antibacterial mechanism of the PMP DN ICH. For PIL, the positively charged imidazole rings interacts with the negatively charged phospholipid bilayer of bacterial cell membrane through electrostatic interaction, thus providing an opportunity for imidazole side chain alkyl to insert into the phospholipid bilayer, resulting in the rupture of bacterial cell membrane and the leakage of cytoplasm, thereby killing the bacteria (Fig. 3b) [42, 43]. Furthermore, studies have shown that oxygen-containing groups of MXene nanosheets can form hydrogen bonds with lipopolysaccharide chains of the bacterial cell membranes, prevent bacteria from ingesting nutrients, inducing bacteria to produce active oxygen components and cell inactivation, thus inhibiting bacterial growth. In addition, the sharp edges of the MXene nanosheets could also enter the cytoplasmic region by cutting the bacterial cell wall, causing the release of bacterial DNA and eventually disintegrate of the bacteria (Fig. 3c) [44].

The antibacterial ability of the PMP DN ICH is shown in Fig. 3d–f. The sizes of the inhibition zones of the PMP DN ICH against *E. coli* and *S. aureus* were found to be 24 and 24 mm (Fig. 3d), respectively, indicating the desirable antibacterial activity of the PMP DN ICH. In addition, *E. coli* and *S. aureus* were incubated with the PMP DN ICH at 37 °C for 18 h. The active colonies of all the investigated microbes were almost vanished (Fig. 3e), and the corresponding antibacterial rates of *E. coli* and *S. aureus* were found to be 99.91% and 99.98%, respectively (Fig. 3f), further displaying the excellent antibacterial property.

### Electrical Sensing Properties of a PMP DN ICH-Based Strain Sensor

**Fig. 4:**
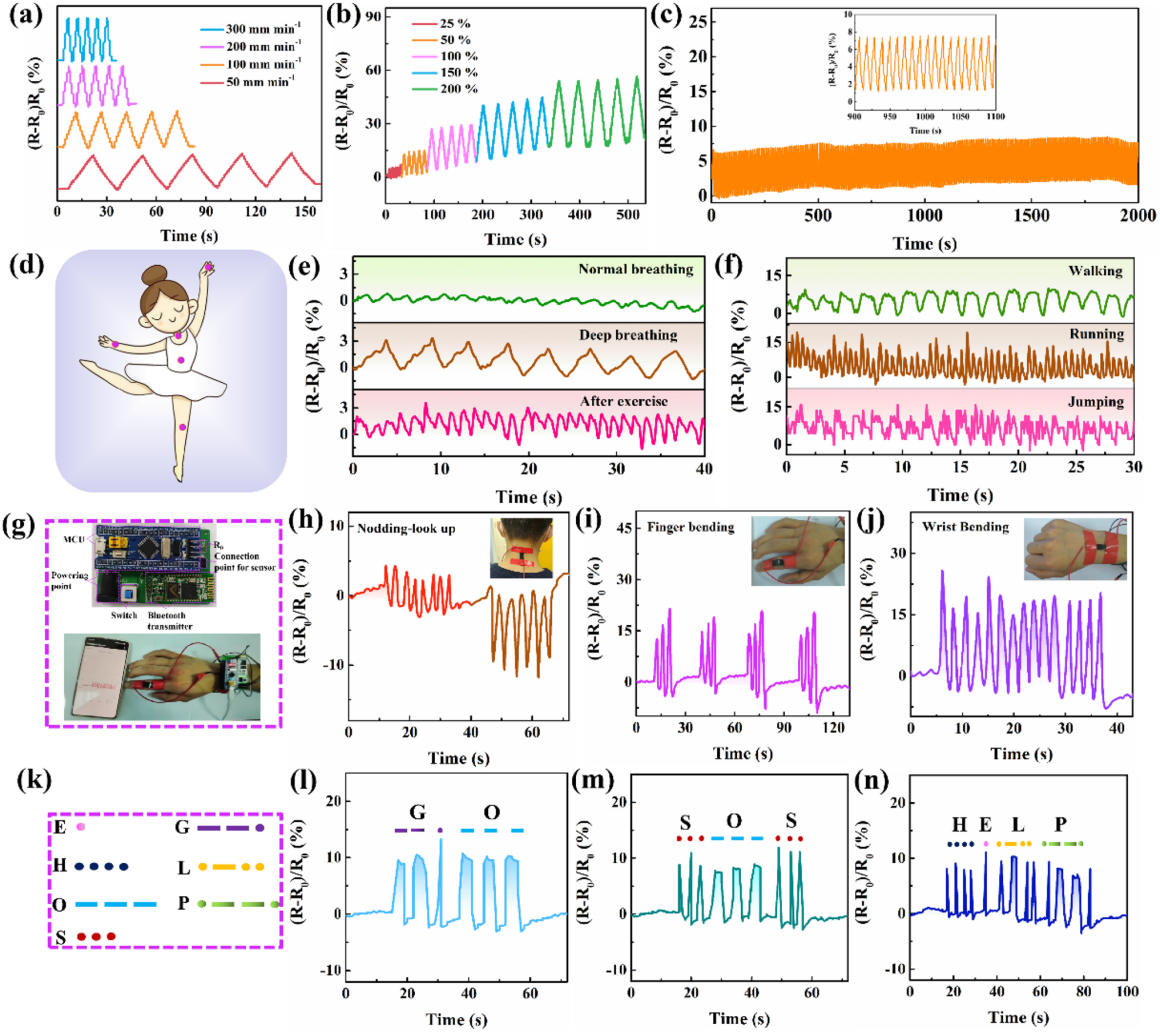
Time-dependent changes in relative resistance of the PMP DN ICH-based strain sensor subjected to cyclic stretching at various **a** tensile rates and **b** tensile strains. **c** Cyclic sensing response of the PMP DN ICH-based strain sensor at 25% strain for ~ 300 stretching cycles (inset: magnified data over a representative timeframe). **d** Schematic of the monitored sensing sites. Variations in relative resistance of the sensor attached to the **e** belly (during normal breathing, deep breathing, and breathing after exercise) and **f** knee (during walking, running, and jumping). **g** Photographs of the wireless transmission device and setup of the practical application. Changes in relative resistance during **h** nodding and looking up movements, **i** finger bending, and **j** wrist bending. **k** Schematic showing the definition of Morse codes. Coding the words **l** “GO,” **m** “SOS,” and **n** “HELP” by short- and long-duration finger bending

To evaluate the potential applicability of the developed ICH in wearable strain sensing, its electrical sensing properties were comprehensively explored by constructing and assessing a PMP DN ICH-based strain sensor. No obvious signal fluctuations were observed at tensile rates ranging from 50 to 300 mm min^−1^ and over a tensile strain range of 25%–200%, which demonstrated the reversible and stable signal output ability of the devised strain sensor (Fig. 4a, b). Moreover, the responsive resistance variation waveforms were consistent with the tensile strain (50%), indicating negligible electromechanical hysteresis (Fig. S16) [45]. The stability of the PMP DN ICH-based strain sensor was tested at a tensile strain of 25% over approximately 300 loading–unloading cycles (Fig. 4c). The relative resistance variation was generally consistent, indicating excellent repeatability and stability of the PMP DN ICH-based strain sensor.

Subsequently, the PMP DN ICH-based strain sensor was attached to different parts of the human body to detect human movement (Fig. 4d). Interestingly, when the sensor was mounted on the belly and knee, it readily detected and distinguished between different breathing and motion states (Fig. 4e, f). Furthermore, to achieve remote monitoring of human movement, a wireless sensing system was constructed by connecting a small portable wireless transmission device to PMP DN ICH (Fig. 4g) [8]. The PMP DN ICH-based strain sensor adhered to the neck or wrist could effectively detect neck nodding, looking up movements, and repetitive wrist motion (Fig. 4h, j and Movie S3). More importantly, the relative resistance increased as the bending angle of the finger increased from 30° to 90°, indicating a high sensitivity (Fig. 4i and Movie S4). Additionally, the ability of the wireless sensing system to convey information using Morse codes was explored (Fig. 4g, k) [46]. Distress signals of “GO,” “SOS,” and “HELP” were encrypted and translated by briefly and extensively stretching the PMP DN ICH-based strain sensor in an alternating manner (Fig. 4l–n and Movies S5, S6, S7). The demonstrations of the PMP DN ICH-based wireless strain sensor present the possibility to use PMP DN ICH as wearable devices for human health monitoring, encrypted transmission of information, and human–machine interfaces.

### Thermal Sensing Properties of a PMP DN ICH-Based Thermistor

**Fig. 5:**
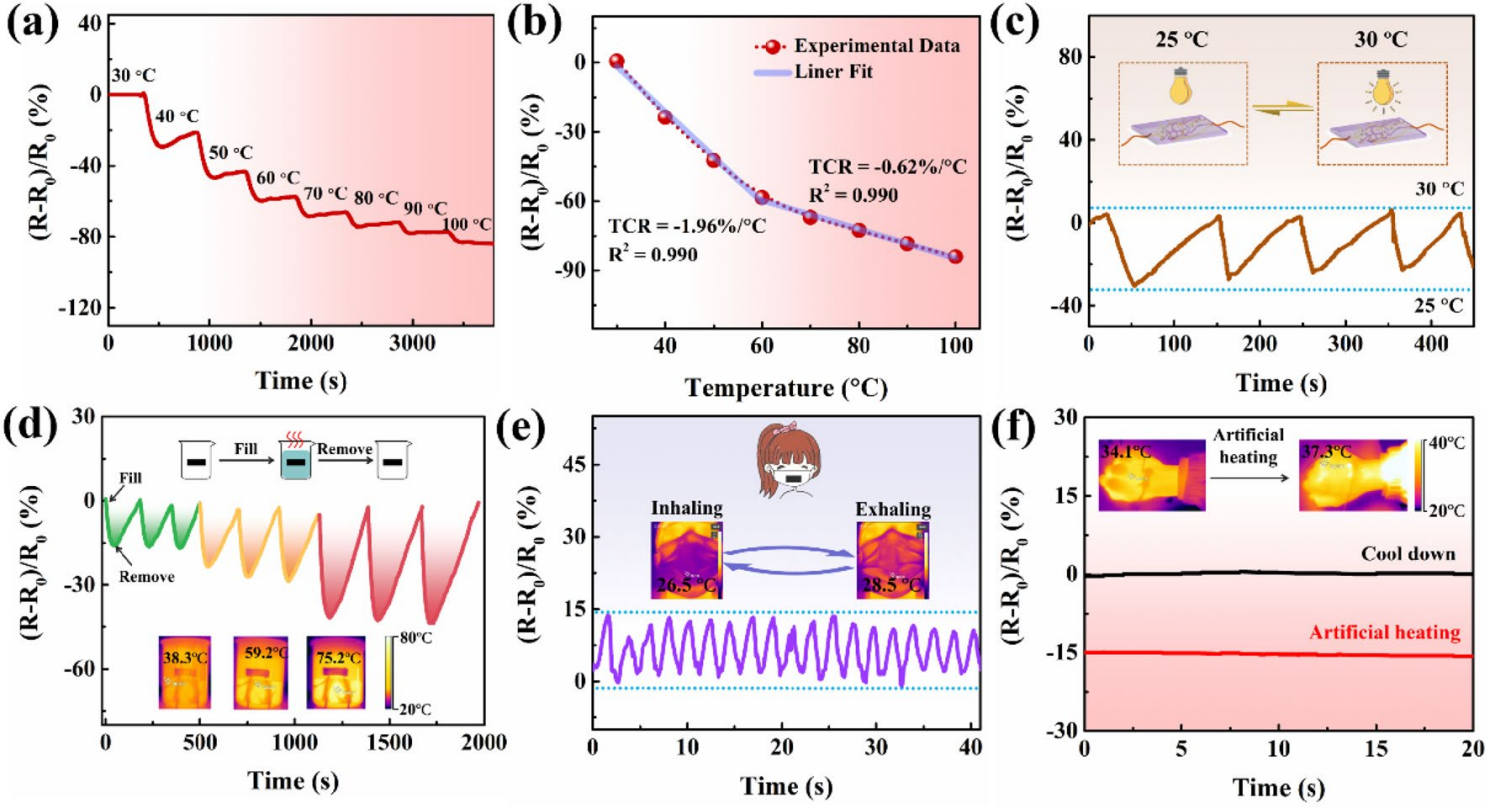
Thermal sensing properties of a PMP DN ICH-based thermistor. Changes in relative resistance of the PMP DN ICH-based thermistor with step increases in temperature from 30 to 100 °C with respect to **a** time and **b** temperature. **c** Dynamic resistance responses of the thermistor during successive heating–cooling cycles between 25 and 30 °C. **d** Dynamic resistance responses of the thermistor to detect the addition or removal of water at different temperatures (38.3, 59.2, and 75.2 °C); inset: infrared images of the added water. **e** Dynamic resistance responses of the thermistor to temperature changes while breathing; inset: the temperature changes of a mask during inhalation and exhalation. **f** Dynamic resistance responses of the thermistor to variations in skin temperature while simulating a fever; inset: changes in the skin temperature before and after the fever

The outstanding temperature resistance of PMP DN ICH was leveraged to use it as a thermosensitive material to explore its viability as a temperature sensor. The thermal sensitivity of a thermistor is commonly evaluated using the temperature coefficient of resistance (TCR), which is estimated using the slope of the fit resistance curves [47]. Therefore, the changes in relative resistance of a PMP DN ICH-based thermistor were monitored with step increases in temperature from 30 to 100 °C (Fig. 5a, b). The obtained TCR values of − 1.96% °C^−1^ (30–60 °C) and − 0.62% °C^−1^ (60–100 °C) (Fig. 5b) are superior to most previously reported TCR data (Table S1), demonstrating the outstanding temperature sensitivity of the PMP DN ICH-based thermistor. Furthermore, the PMP DN ICH-based thermistor demonstrated excellent repeatability of the thermal response over relatively small and large temperature ranges (Fig. 5c, d). These results indicated that the PMP DN ICH-based thermistor had excellent thermal sensitivity and could be applied as a thermal sensor for monitoring environmental temperature changes. More importantly, the fabricated thermistor showed remarkable potential in quantitatively monitoring the human body temperature (Fig. 5e, f). For example, the thermistor was fixed onto a mask to detect normal breathing by monitoring the temperature changes during exhalation and inhalation (Fig. 5e). Additionally, when an external heat source was used to simulate the temperature of human fever (Fig. 5f), the thermistor effectively responded to changes in the body temperature through variations in its relative resistance. The above results indicate that the PMP DN ICH-based thermistor is suitable for monitoring the changes in ambient and body temperatures.

### Electrochemical Properties of a Flexible PMP DN ICH-Based All-Solid-State Supercapacitor

**Fig. 6:**
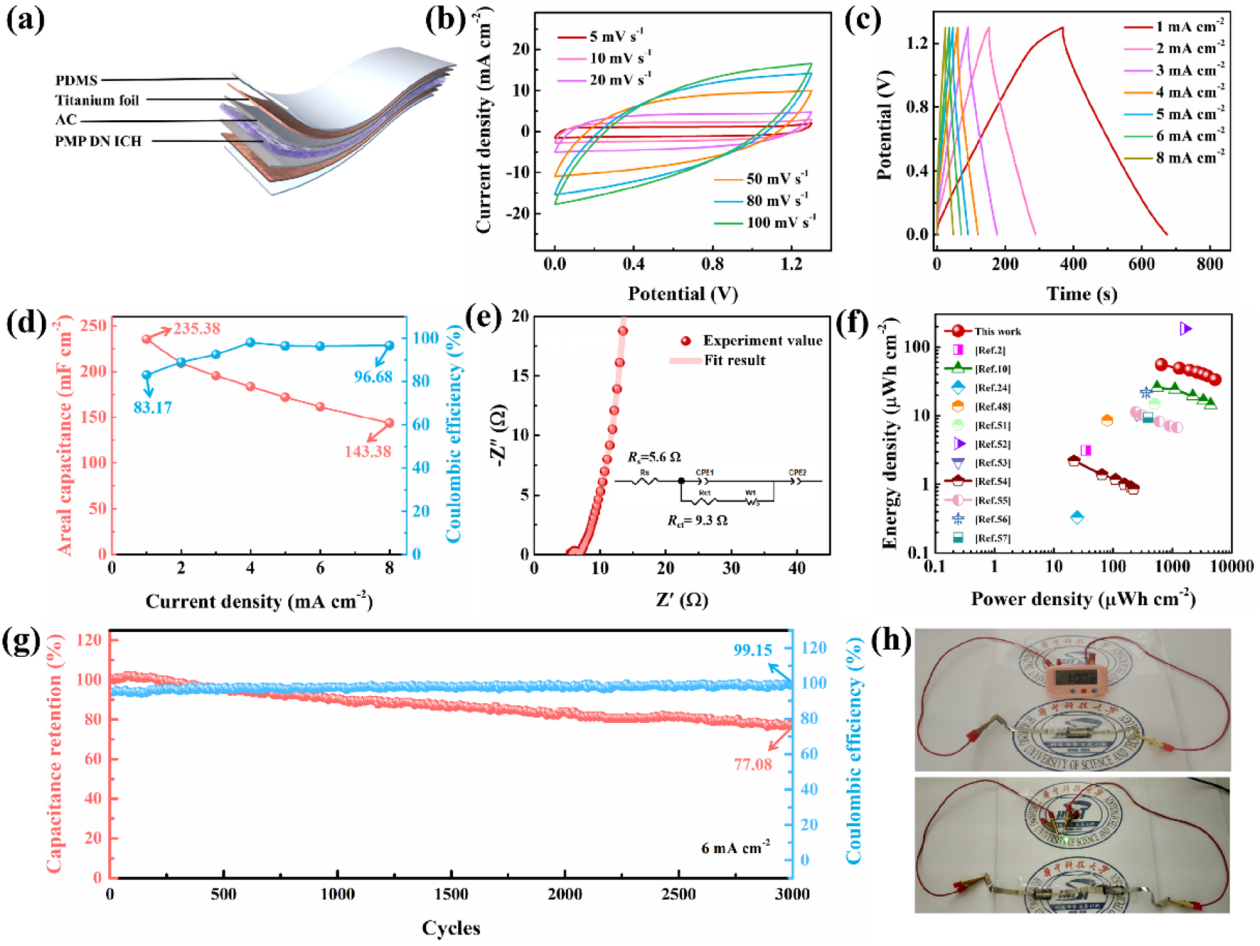
Electrochemical properties of the PMP DM ICH-based SC. **a** Illustration of the assembled SC. **b** CV curves of the fabricated SC at scan rates of 5–100 mV s^−1^). **c** GCD curves and **d** the corresponding areal capacitance and coulombic efficiency of the SC at current densities of 1–8 mA cm^−2^. **e** EIS curve and the corresponding equivalent circuit diagram of the devised SC. **f** Ragone plot comparing the energy and power densities of the SC with those reported previously. **g** Cycling stability of the SC at 6 mA cm^−2^. **h** Photographs illustrating the ability of the SC to power an electronic meter and LED bulb

To certify the ability of PMP DN ICH to effectively function as an electrolyte, given its unique advantages, a flexible all-solid-state SC was assembled by combining the PMP DN ICH electrolyte exhibiting adhesive properties with an activated carbon electrode (Fig. 6a). The electrochemical performance of the flexible PMP DN ICH-based SC was evaluated by cyclic voltammetry (CV), galvanostatic charge–discharge (GCD) analysis, and EIS. The optimal working potential window of the fabricated SC was determined to be 1.3 V (Fig. S17). As shown in Fig. 6b, the shapes of CV curves were close to rectangle as the scan rate increased from 5 to 100 mV s^−1^ over the working potential window of 0–1.3 V, exemplifying the typical electronic double layer capacitive performance and excellent rate capability [24, 48]. GCD curves of the SC obtained at various current densities (1–8 mA cm^−2^) displayed symmetric triangular shapes and featured a small IR drop even at a high current density of 8.0 mA cm^−2^, indicating that the SC exhibited nearly ideal charge–discharge ability and decent capacitive behavior (Fig. 6c) [49]. Notably, even at the highest imposed current density of 8 mA cm^−2^, the areal capacitance and coulombic efficiency of the SC were maintained at 143.38 mF cm^−2^ and 96.68%, respectively (Fig. 6d). These values are considerably higher than those obtained in previous hydrogel-based studies (Table S2). In addition, the PMP DN ICH-based SC possesses a wide electrochemical window and a range of operating temperatures, which ensure that the SC can be used normally in harsh environments. The EIS curve and the corresponding equivalent circuit of the SC (Fig. 6e) were used to determine its equivalent series resistance (*R*_s_) and charge-transfer resistance (*R*_ct_) from the intercepts of the approximate semicircular area in the high-frequency region of the Nyquist plot [25, 30]. The low *R*_s_ (5.6 *Ω*) and *R*_ct_ (9.3 *Ω*) values indicate good electrode–electrolyte contact and the occurrence of efficient charge transfer. Moreover, the data in the low-frequency region were almost parallel to the *Z*′′-axis, demonstrating the excellent capacitive behavior of the fabricated SC [50]. The Ragone plot suggests that the maximum energy and power densities of the devised SC (55.25 μWh cm^−2^ and 5200 μWh cm^−2^, respectively) are superior to those of most reported hydrogel-based SCs (Fig. 6f) [2, 10, 24, 48, 51–57]. Furthermore, the fabricated SC retained an initial areal capacitance of 77.08% and an almost unchanged coulombic efficiency of 99.15%, indicating its decent long-term stability (Fig. 6g) [58]. The practical viability of the fabricated SC was assessed by using it to power small electronic devices (Fig. 6h). The SC with a volume of 2 × 1 × 0.3 cm^3^ was able to drive an electronic meter for 3 min (Movie S8), whereas two SCs connected in series could illuminate an LED bulb for 47 s (Movie S9). These electrochemical results highlight the broad application prospects of the PMP DN ICH-based SC in developing flexible energy-storage systems for wearable electronics.

### Electrochemical Performance of the PMP DN ICH-Based SC Under Different Conditions

**Fig. 7:**
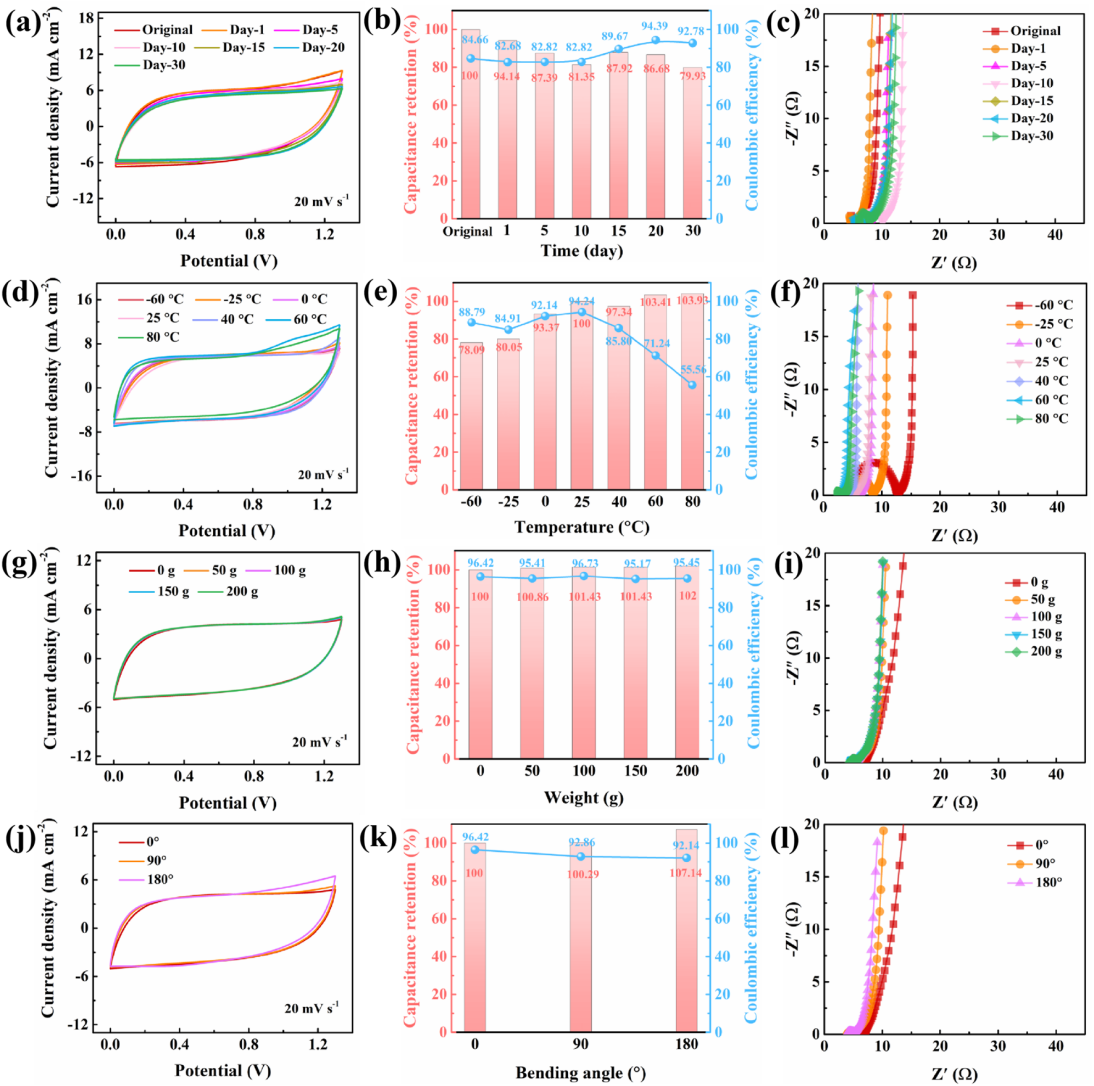
CV curves (scan rate, 20 mV s^−1^); capacitance retention and coulombic efficiency (current density, 6 mA cm^−2^); and EIS curves of a PMP DN ICH-based SC for different **a**–**c** storage durations, **d**–**f** temperatures, **g**–**i** pressures, and **j**–**l** bending angles

PMP DN ICH was found to exhibit outstanding mechanical performance, excellent temperature resistance, and long-term stability. Therefore, the electrochemical performance of the PMP DN ICH-based SC was meticulously probed under various conditions (Figs. 7 and S18). The CV curves nearly overlapped as the storage duration increased to 30 d (Fig. 7a). Moreover, the final capacitance retention and coulombic efficiency were 79.93% and 92.78%, respectively (Figs. 7b and S18a), and the Nyquist plots shifted slightly to the right (Fig. 7c), demonstrating the remarkable long-term stability of the SC. The temperature resistance of the SC was investigated by increasing the temperature from − 60 to 80 °C. The CV integration area increased compared to that of the initial state (Fig. 7d), and the *R*_s_ value from the Nyquist plots decreased (Fig. 7f) owing to the enhanced ionic diffusion efficiency of the SC at elevated temperatures. Furthermore, the capacitance retention increased gradually from 78.09% to 103.93%, and the coulombic efficiency decreased from 88.79% to 55.56% as the temperature increased from − 60 to 80 °C (Figs. 7e and S18b). The possible reason for this behavior is that with the increasing temperature, the reaction kinetics of some side reactions in the charging process of the supercapacitor accelerated, and the side reaction was prompted to occur at a high temperature, thus extending the charging time of the supercapacitor, resulting in the reduction of its coulombic efficiency [59]. Subsequently, the electrochemical stability of PMP DN ICH under different mechanical stimuli was studied (Figs. 7g–l and S18c, d). Interestingly, the CV curves and Nyquist plots nearly overlapped for different loadings as well as bending angles (Fig. 7g, i, j, l). Moreover, the capacitance retention and coulombic efficiency remained almost unchanged (Figs. 7h, k and S18c, d), indicating the stable energy-storage characteristics of the SC. Overall, the excellent environmental stability, temperature tolerance, and mechanical flexibility of the SC enabled it function as a reliable power source at different temperatures and under varying mechanical conditions. This high-performance PMP DN ICH electrolyte paves a new way for flexible and wearable SCs and could have a wide range of applications in advanced energy-storage devices.

### Electrical Output Properties of a PMP DN ICH-Based Triboelectric Nanogenerator

**Fig. 8:**
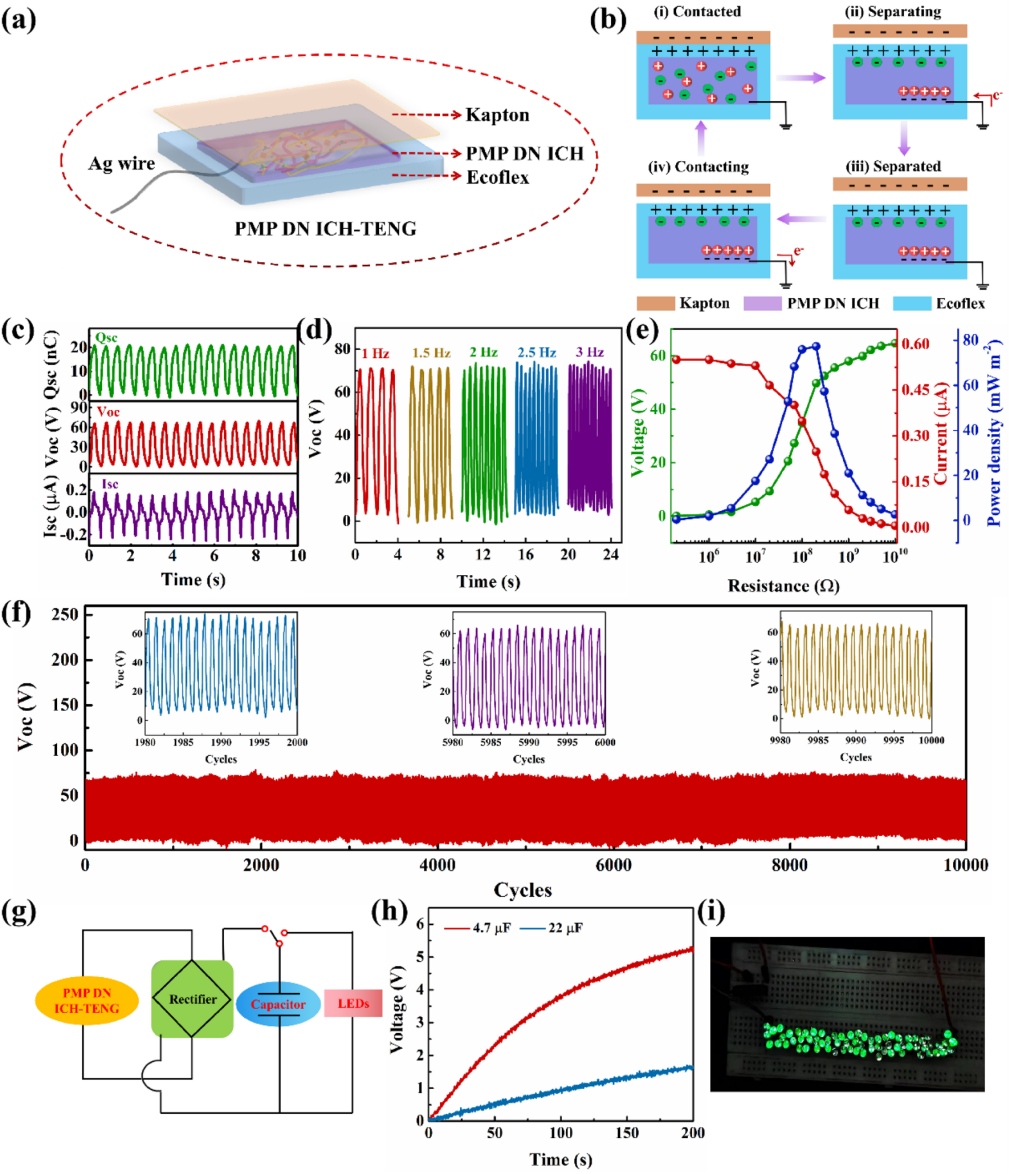
Schematics of the **a** PMP DN ICH-based TENG and its **b** working principle. **c** Output *V*_oc_, *I*_sc_, and *Q*_sc_ values of the TENG. **d** *V*_oc_ values of the TENG for different frequencies (1–3 Hz). **e** Electrical output properties of the TENG with different external resistors. **f** Long-term stability test of the TENG. **g** Equivalent circuit diagram of a self-charging power system based on the TENG. **h** Charging behavior of capacitors (4.7 and 22 μF) at a working frequency of 2 Hz. **i** Photograph of 68 commercial green LEDs illuminated by the TENG

PMP DN ICH was subsequently used as a current collector to assemble a single-electrode-mode TENG (Fig. 8a). An elastomeric silicone rubber substrate (Ecoflex 00–50) was employed as the positive friction layer, a commercial Kapton film was used as the negative-contact triboelectric material layer, and Ag wire was adopted as the electrode [3]. The operating mechanism of the PMP DN ICH-based TENG is illustrated in Fig. 8b. When the Kapton film is separated from the silicone rubber layer, no electric potential is present between the films. However, when the Kapton film touches the TENG, electrons are transferred from the silicone rubber layer to the Kapton film, yielding a positively and negatively charged silicone rubber layer and Kapton film, respectively (Fig. 8b(i)). When the Kapton film is separated and removed, PMP DN ICH provides a negative charge to compensate for the positive charge on the surface of the silicone rubber layer, leading to electron flow from the external circuit to PMP DN ICH (Fig. 8b(ii)). Subsequently, an electrostatic equilibrium is achieved when the Kapton film and silicone rubber layers are completely detached (Fig. 8b(iii)). Once the Kapton film is reconnected to the TENG, electrons are repelled from the PMP DN ICH electrode to ground (Fig. 8b(iv)). Finally, an alternating current (AC) electric signal is generated through continuous contact-separation events.

Typical electrical output measurements of a standard PMP DN ICH-based TENG (40 × 40 mm^2^; frequency, 2 Hz) were performed under a force load of 2 N. The open-circuit voltage (*V*_oc_), short-circuit current (*I*_sc_), and transferred short-circuit charge (*Q*_sc_) of the devised TENG were 66.0 V, 0.18 μA, and 20.6 nC, respectively (Fig. 8c). The electrical output performance of the PMP DN ICH-based TENG under various frequencies (1–3 Hz) was measured under a force load of 2 N (Fig. 8d). It was found that the *V*_oc_ remained relatively stable and reached 67.6 V. Moreover, the TENG could drive loads under different external resistances of 10^5^–10^10^ Ω (Fig. 8e), with the optimal output power density being 77.3 mW m^−2^ at a load resistance of 2 × 10^8^ Ω. The output voltage stability of the TENG was then monitored for 10,000 contact-separation cycles at a frequency of 2 Hz (Fig. 8f). The superior electrical output reliability indicated that the TENG could satisfy practical application requirements. Subsequently, the TENG was connected to external capacitive loads and LEDs in a commercial rectifier circuit (Fig. 8g). Furthermore, the ability of the TENG to be continuously charged was analyzed at different capacitances (Fig. 8h). Notably, the charging speed accelerated with decreasing capacitance, with the 4.7 and 22 µF capacitors achieving voltages of 5.2 and 1.6 V, respectively, by tapping the device for 200 s at a frequency of 2 Hz. Additionally, the charging ability of the TENG as a power source for real-time practical applications was further assessed by illuminating 68 green commercial LEDs (Fig. 8i and Movie S10), underscoring the significant application potential of the devised TENG for low-frequency mechanical energy harvesting.

## Conclusion

A PMP-based DN ICH with excellent temperature resistance, acceptable mechanical properties, outstanding conductivity, long-term stability, high oxidation resistance, and antibacterial activity was successfully prepared using freeze–thawing and ionizing radiation technology. Experimental results demonstrated the high sensitivity, fast response ability, and excellent sensing stability of PMP DN ICH; consequently, the ICH was applied to human motion monitoring and thermal sensing to probe environmental temperature changes. The all-solid-state SC based on PMP DN ICH operated adequately during prolonged storage as well as at various temperatures and under different mechanical stimuli. More importantly, the single-electrode PMP DN ICH-based TENG exhibited favorable energy-harvesting performance as a self-charging power system. These applications illuminate that this work provides an important approach to construct high-performance ICH for multifunctional/flexible wearable sensing, energy-storage, and energy-harvesting technologies. In addition, the PMP DN ICH may have broad application prospects in smart wearable devices, human–machine, and advanced energy-storage devices.

## Supplementary Information

Below is the link to the electronic supplementary material.

Supplementary file 1 (ZIP 235812 kb)

Supplementary file 2 (PDF 2304 kb)
