# Supplementary material for: An Environment-Tolerant Ion-Conducting Double-Network Composite Hydrogel for High-Performance Flexible Electronic Devices
Source: Nanomicro Lett. 2024 Jan 29;16:99. doi: 10.1007/s40820-023-01311-2 (PMC10825113; doi:10.1007/s40820-023-01311-2)
Supplement: Supplementary file 2 — (PDF 2304 kb) [file 40820_2023_1311_MOESM2_ESM.pdf]

Supporting Information for

An Environment–Tolerant Ion–Conducting Double–Network Composite Hydrogel for High–Performance Flexible Electronic Devices

Wenchao Zhao[1,2], Haifeng Zhou[1,2], Wenkang Li[1,2], Manlin Chen[1], Min Zhou[1], and Long Zhao[1,]*

[1] State Key Laboratory of Advanced Electromagnetic Technology, School of Electrical and Electronic Engineering, Huazhong University of Science and Technology, Wuhan 430074, P. R. China

[2] School of Chemistry and Chemical Engineering, Huazhong University of Science and Technology, Wuhan 430074, P. R. China

*Corresponding author. E–mail: zhaolong@hust.edu.cn (Long Zhao)

S1 Experimental Section

S1.1 Synthesis of Ph–3MVIm–Br

Firstly, 1,3,5–tris(bromomethyl)benzene (12.00 g, 33.0 mmol), 1–vinylimidazole (10.34 g, 108.7 mmol) and BHT (0.60 g, 2.70 mmol) were dissolved in CH_3CN (300 mL). The above solution was rigorously stirred for 60 h at 338 K. The crude product was filtered and dissolved in water. Then, the aqueous solution was evaporated under reduced pressure at 323 K. Subsequently, the resultant monomer was dried under high vacuum at 348 K. The results of ^1H NMR spectra for Ph–3MVIm–Br was shown in Fig. S1.

S1.2 Exploration and Optimization of Radiation Synthesis Conditions of the PMP DN ICH

The gel fraction (GF) and conductivity were shown in Figs. S2 and S3, respectively. The GF represents the mass fraction of the cross–linked content of the gel system that cannot be dissolved by the relevant solvent [S1]. As shown in Fig. S2a, the GF of the PMP DN ICH increased with the absorbed dose, while the conductivity decreased with the absorbed dose (Fig. S3a). Higher GF typically implies a higher crosslinking density and a denser network. The denser network hinders ion transport, thus decreasing the conductivity [S2]. According to the results of GF and conductivity, the optimal absorbed dose of the PMP DN ICH was determined to be 20 kGy. This results

also explains the increase in GF and decrease in conductivity with the increasing monomer (VBImBr) concentration (Figs. S2b and S3b) and the crosslinker (Ph–3MVIm–Br) concentration (Figs. S2c and S3c), respectively. Figs. S2d and S3d shown the GF and conductivity of the PMP DN ICH as a function of the MXene contents. With an increase in the MXene contents, the GF increased, while the conductivity first increased and then decreased. The abundant –OH functional groups on the surface of MXene formed many hydrogen bonds in the gels, which increased the crosslinking density [S3]. Additionally, a well–connected MXene network can be formed to enhance the ion transport capacity of the gels [S4]. However, the content of MXene in the system was excessive for dispersion, which is detrimental to the conductivity of the gels [S5].

In summary, the optimal synthesis conditions of the PMP DN ICH were determined as follows: absorbed dose of 20 kGy, monomer concentration of 8 mol L^{-1}, crosslinker concentration of 0.02 mol L^{-1}, and MXene content of 1.5 wt.%. The gel synthesized under these conditions was used in the subsequent experimental investigation.

S1.3 Gel Fraction Tests

The irradiated PMP DN ICH sample was dried in a vacuum. The gel fraction was estimated gravimetrically through the measurement of the insoluble parts of the samples after extraction in H_2O for 5 days, then calculated as follows:

$$Gel\ fraction = (W_g / W_0) \times 100\% \qquad \text{(S1)}$$

Where the W_g was the weight of the gel part and the W_0 was the original total dry weight. The samples were tested in parallel in three groups.

S1.4 Temperature Resistance

The temperature resistance property of the PMP DN ICH at various temperature (−60, −25, 0, 25, 40, 60, and 80 °C) were immediately evaluated after the PMP DN ICH was storage at various temperature for 4 h. The samples were tested in parallel in three groups.

S1.5 Durable Stability Tests

The stability behavior of the PMP DN ICH were evaluated by water retention (WR) at different times (at room temperature, 40%–60% relative humidity). The weight changes of the PMP DN ICH were measured and then compared with the original weight. The samples were tested in parallel in three groups. The WR was calculated by the following equation (S2):

$$WR = (W - W_0)/W_0 \times 100\% \qquad \text{(S2)}$$

Where W and W_0 represent the weight after storage and the original weight, respectively.

S1.6 Oxidation Stability Test of MXene in the PMP DN ICH

The proportion of each substance in the MXene + H_2O + PVA + ILs solution was as follow: 27.72 mg MXene: 0.9 g H_2O: 0.1 g PVA: 1.848 g monomer: 12.77 mg cross–linker. For MXene + H_2O and MXene + H_2O + PVA solutions, Refer to the above steps for the proportion of components. Then, the gels under the appropriate conditions were prepared by freeze-thaw or further irradiation technology.

The antioxidant stability of MXene in the above solutions were tested by adding the same volume of H_2O_2 (30%, 0.5 mL) solution to the above solutions. For gels, the oxidation state of MXene at different times was recorded after the gels were soaked in H_2O_2 (30%) solution and removed.

S1.7 In Vitro Antibacterial Activity Tests

The antibacterial properties evaluation of the PMP DN ICH were measured according to the preliminary work [S6]. The antibacterial ability was evaluated by the following equation (S3):

$$\text{Antibacterial rate} = \left(1 - \text{bacterial count of samples} / \text{bacterial count of negtive control}\right) \times 100\% \qquad \text{(S3)}$$

S1.8 Areal Capacitance

The area specific capacitance C_A (mF cm^{-2}) of the PMP DN ICH-based SC was calculated from the GCD curves as following:

$$C = I \times \Delta t / (A \times \Delta V) \qquad \text{(S4)}$$

I (mA): the constant charge/discharge current; Δt (s): the discharge time; A (cm^2): the surface total area of the AC electrode; ΔV (V): the voltage window during the discharge progress.

S1.9 Energy Density and Power Density

The energy density (E, μWh cm^{-2} and power density (P, μW cm^{-2} of the SC were calculated according to the following equations:

$$E_A = C_A \times \Delta V^2 / (2 \times 3600) \qquad (S5)$$

$$P_A = E_A \times 3600 / \Delta t \qquad (S6)$$

S2 Supplentary Figures and Tables

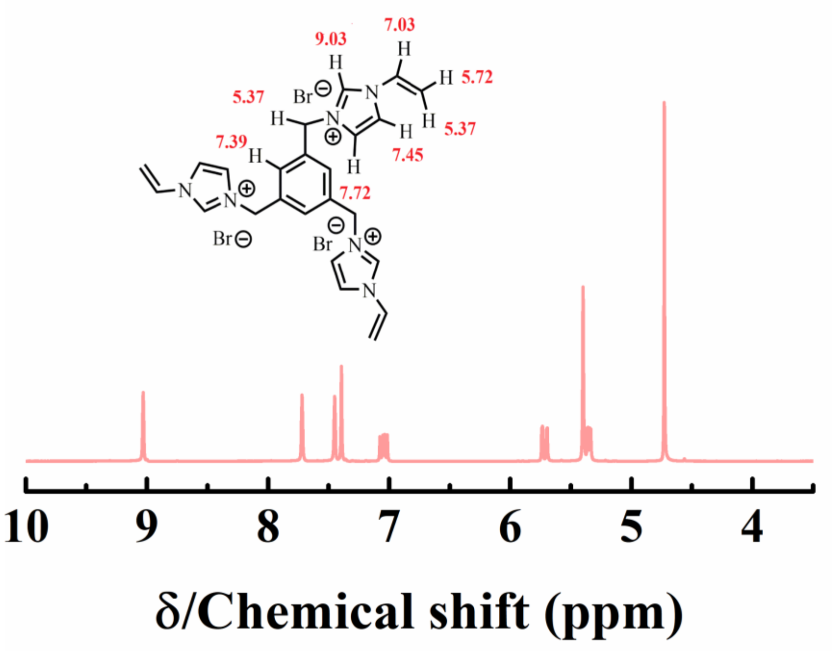

Fig. S1 ^{1}H NMR spectra of Ph–3MVIm–Br in D_2O

Ph–3MVIm–Br: ^{1}H NMR δH (400 MHz, D_2O, ppm) δ 7.72 (d, 3H, –N–CHCH–N–), 7.45 (d, 3H, –NCHCH–N–), 7.39 (s, 3H, –Ph–$(CH_2)_3$–), 7.03 (dd, 3H, –CH=CH_2), 5.72 (dd, 3H, –CH=CH_2), 5.37 (s, 6H, –Ph–$(CH_2)_3$–), 5.37 (dd, 3H, –CH=CH_2).

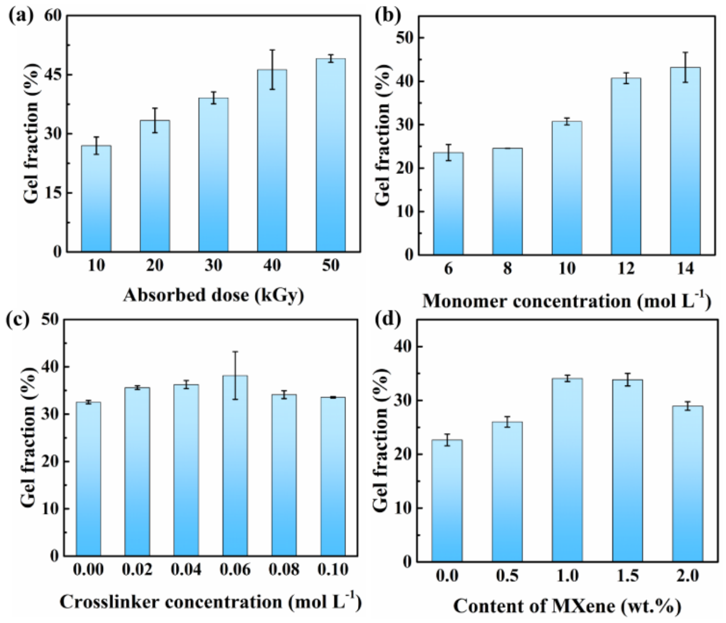

Fig. S2 The gel fraction of the PMP DN ICH as a function of: **a** Absorbed dose (the concentration of the monomer and cross–linker were 10 mol L^{-1} and 0.02 mol L^{-1}, respectively, and the MXene content was 1.0 wt.%). **b** Monomer concentration (the absorbed dose was 20 kGy; the cross–linker concentration was 0.02 mol L^{-1}, and the MXene content was 1.0 wt.%). **c** Cross–linker concentration (the absorbed dose was 20 kGy; the monomer concentration was 8 mol L^{-1}, while the MXene content was 1.0 wt.%). **d** Different contents of MXene (the absorbed dose was 20 kGy; the monomer concentration was 8 mol L^{-1}, while the cross–linker concentration was 0.02 mol L^{-1}). The error bars represent standard deviation; sample size n = 3

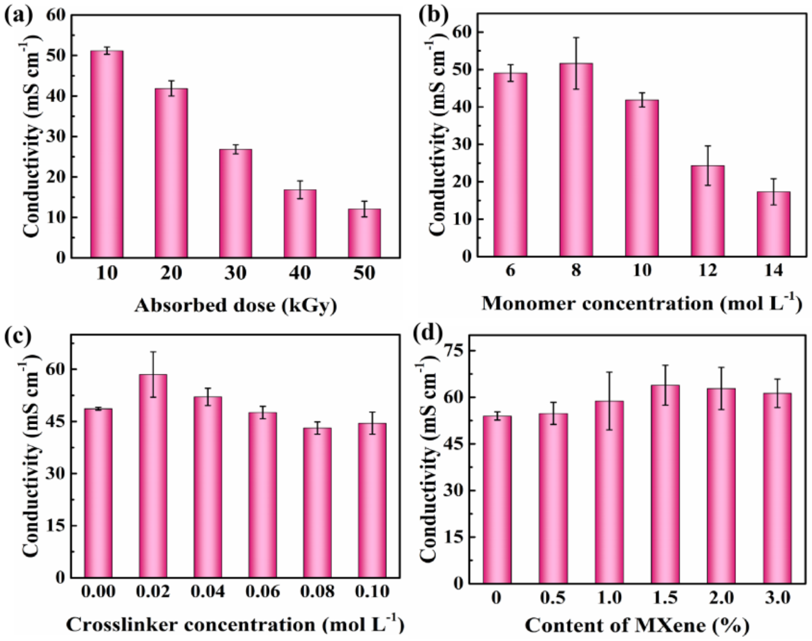

Fig. S3 Conductivity of the PMP DN ICH as a function of: **a** Absorbed dose (the

concentration of the monomer and cross–linker were 10 mol L^{-1} and 0.02 mol L^{-1}, respectively, and the MXene content was 1.0 wt.%). **b** Monomer concentration (the absorbed dose was 20 kGy; the cross–linker concentration was 0.02 mol L^{-1}, and the MXene content was 1.0 wt.%). **c** Cross–linker concentration (the absorbed dose was 20 kGy; the monomer concentration was 8 mol L^{-1}, while the MXene content was 1.0 wt.%). **d** Different contents of MXene (the absorbed dose was 20 kGy; the monomer concentration was 8 mol L^{-1}, while the cross–linker concentration was 0.02 mol L^{-1}). The error bars represent standard deviation; sample size n = 3

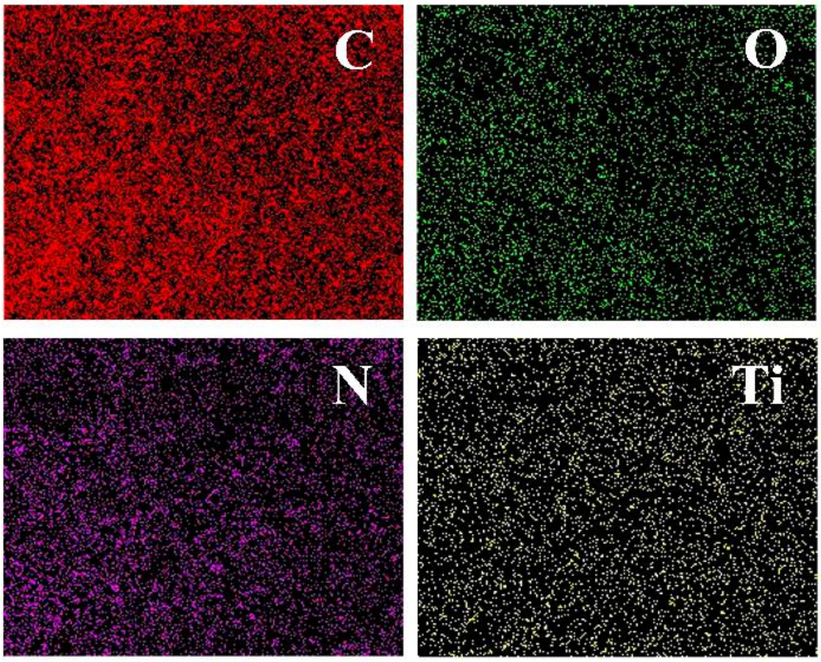

Fig. S4 EDS spectra of the PMP DN ICH

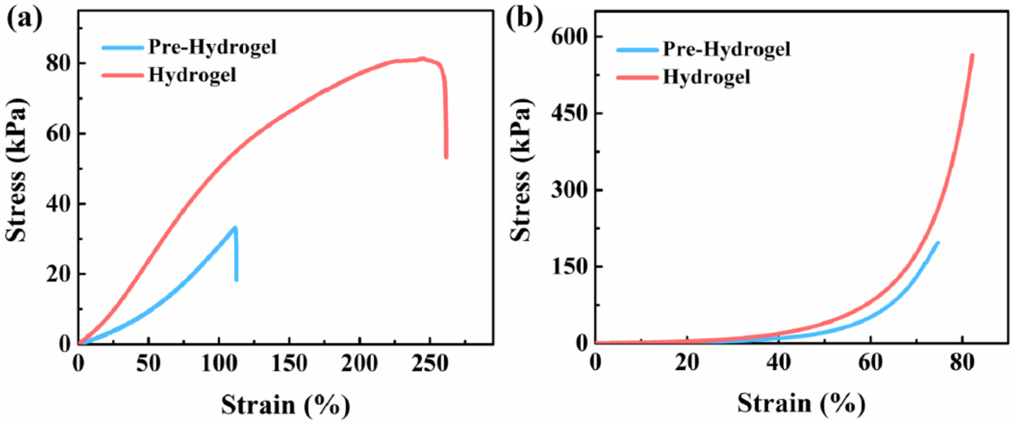

Fig. S5 Comparison the tensile **a** and compressive **b** stress–strain curves of the Pre–PMP DN ICH and PMP DN ICH

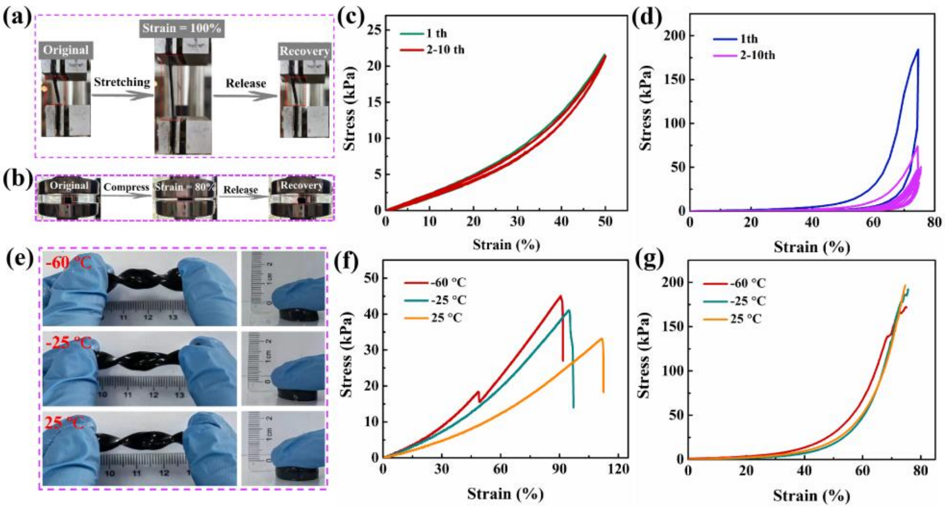

Fig. S6 **a**, **b** Photos of the Pre–PMP DN ICH undergoing stretching and compression. **c**, **d** Cyclic tensile and compressive loading–unloading curves of the PMP DN ICH at strains of 50% and 70% for 10 successive cycles. **e** Photographs showing the temperature tolerance behaviors of the Pre–PMP DN ICH. **f**, **g** Tensile and compressive stress–strain curves for the Pre–PMP DN ICH from −60 to 25 °C

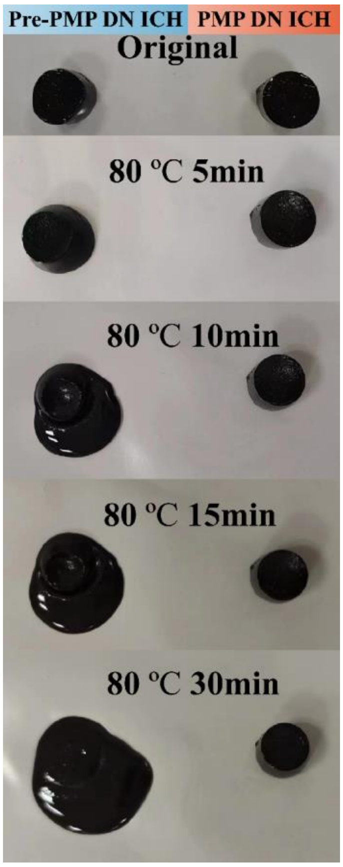

Fig. S7 Comparison of the Pre–PMP DN ICH (left) and PMP DN ICH (right) at 80 °C for different storage time

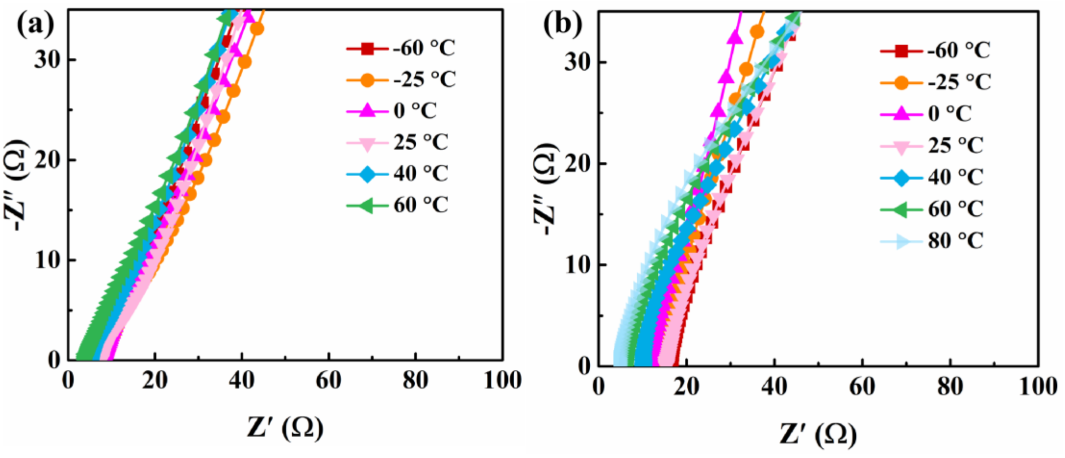

Fig. S8 Nyquist plots of Pre–PMP DN ICH **a** and PMP DN ICH **b** at different temperature

The Fig. S8 shown the EIS curves of the Pre–PMP DN ICH and PMP DN ICH at different temperature. All curves presented a linear trend, which proved that the ion conduction within the DN ICH was a non–Faradic process, no redox reaction occurred, and with no matter or charge crossing the electrode–conductor interface [S7].

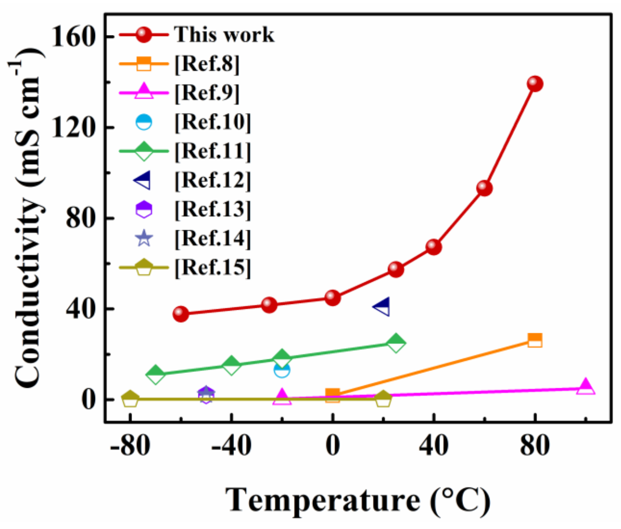

Fig. S9 Conductivity as a function of the temperature of PMP DN ICH in comparison with previously reported temperature tolerance hydrogels [S7–S14]

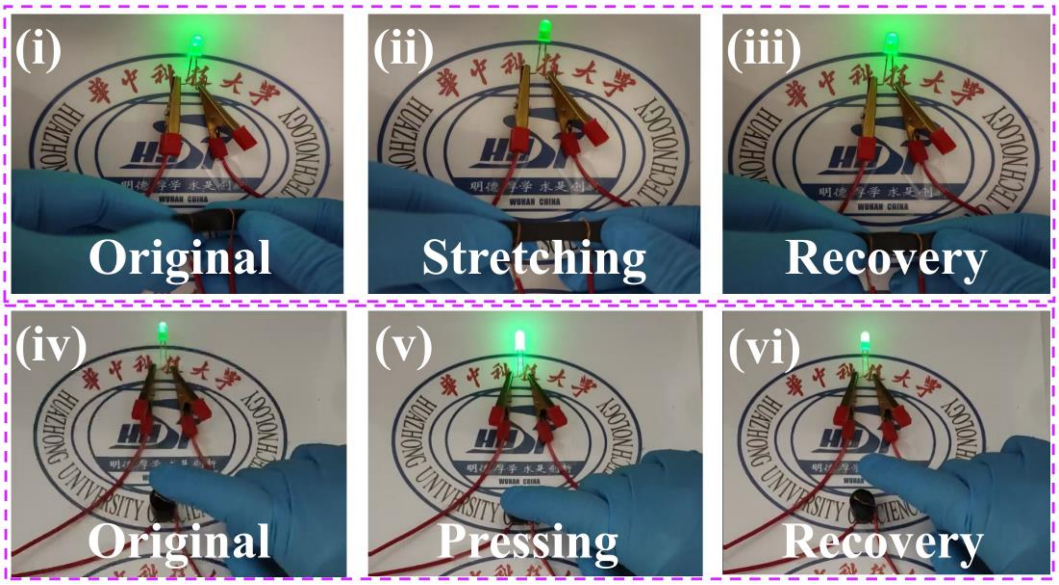

Fig. S10 Comparisons the luminance of LEDs (working voltage of 3.0 V) by using PMP DN ICH as conductor at different states

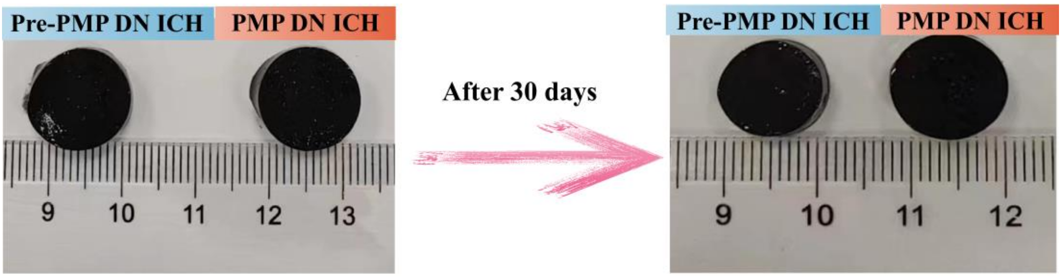

Fig. S11 Photos showing of Pre–PMP DN ICH and PMP DN ICH on the original and 30 d

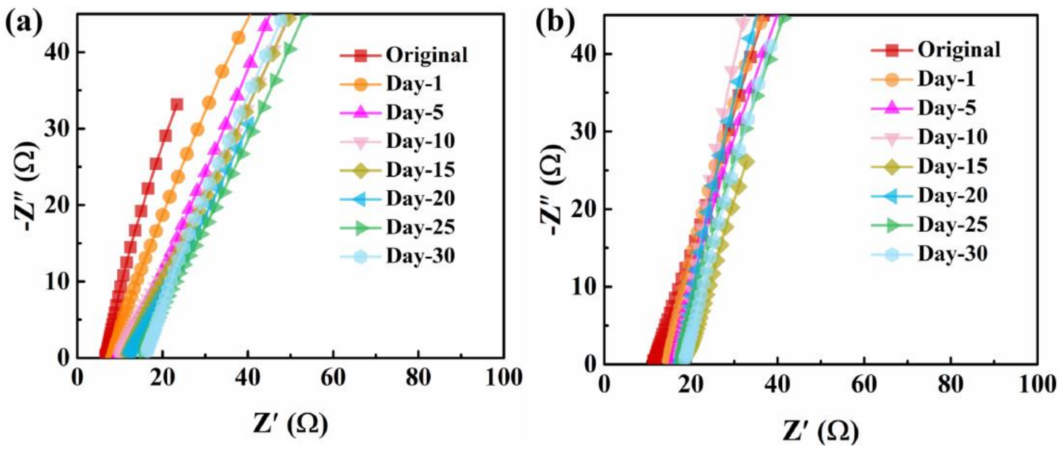

Fig. S12 Nyquist plots of Pre–PMP DN ICH **a** and PMP DN ICH **b** at different storage days

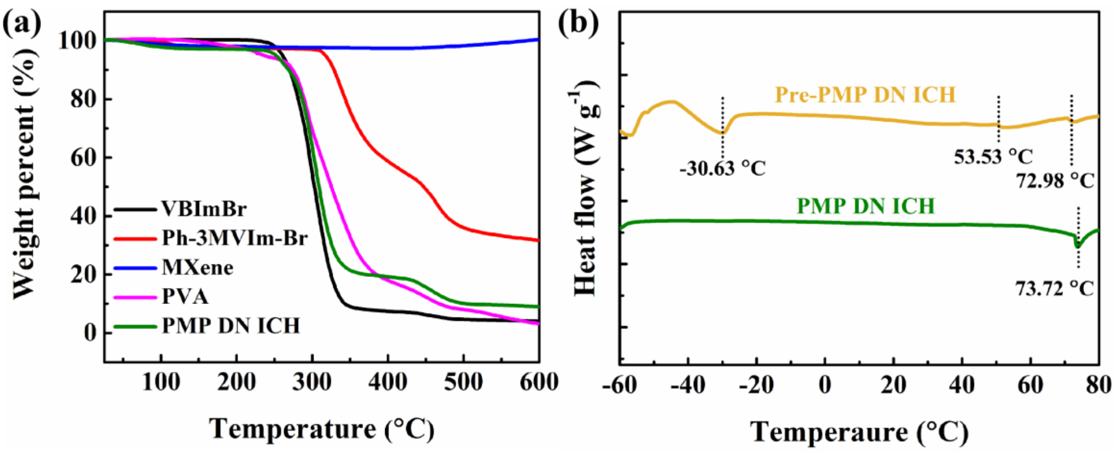

Fig. S13 a TGA patterns of VBImBr, Ph–3MVIm–Br, MXene, PVA, and PMP DN ICH, respectively. **b** DSC curves of Pre–PMP DN ICH and PMP DN ICH

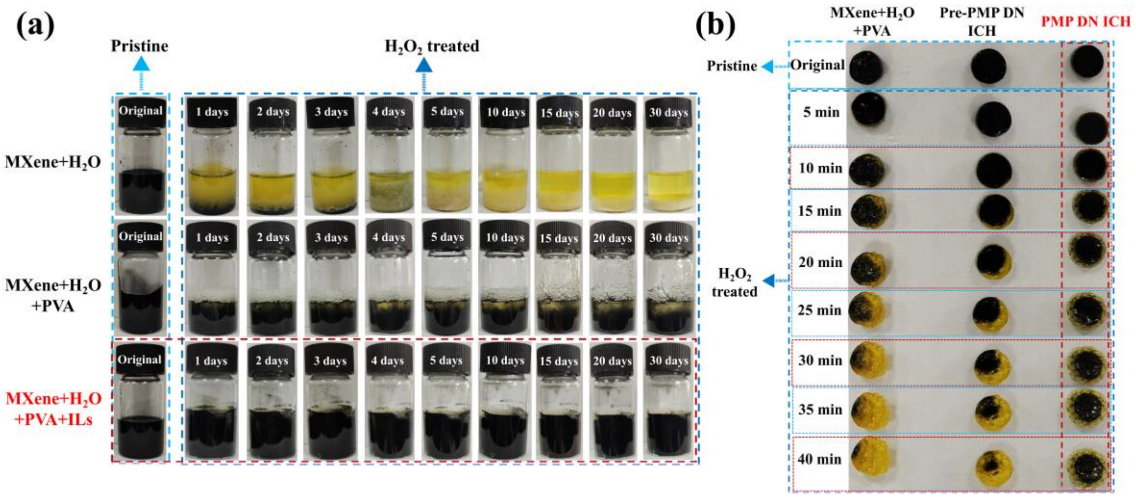

Fig. S14 a Photo showing of MXene + H_2O, MXene + H_2O + PVA, and MXene + H_2O + PVA + ILs solutions during their reaction with H_2O_2 for 30 days. **b** Digital images showing the H_2O_2 interaction effect on MXene + H_2O + PVA hydrogel, Pre–PMP DN ICH, and PMP DN ICH

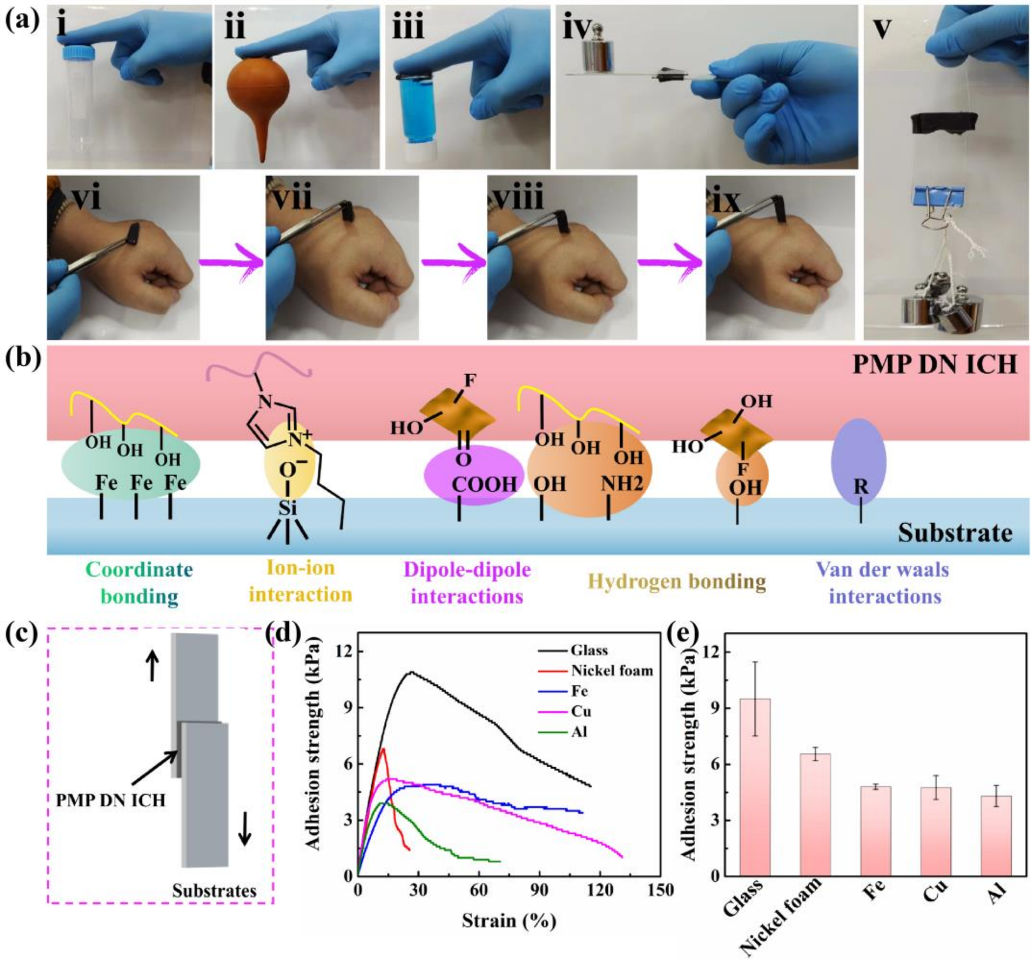

Fig. S15 Adhesion properties of the PMP DN ICH. **a** Adhesive behavior of the PMP DN ICH adhering to different substrates. **b** Adhesion mechanisms between the PMP DN ICH and the varying substrates. **c** Schematic diagram of the adhesion testing process of the PMP DN ICH. **d**, **e** Adhesion strength of PMP DN ICH adhering to different substrates of glass, nickel foam, Fe, Cu, and Al. The error bars represent standard deviation; sample size n = 3

As expected, the PMP DN ICH also exhibited good adhesiveness to different substrates (Fig. S15), which was primarily ascribed to the abundant intermolecular forces (e.g., hydrogen bond, coordinate bond, ion–ion interaction, dipole–dipole interactions, and van der waals interactions) among the component [S9, S15].

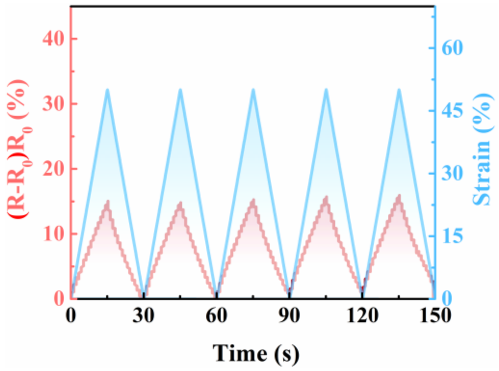

Fig. S16 The relative resistance changes with the tensile strain of 50% during the five continuous loading–unloading cycles

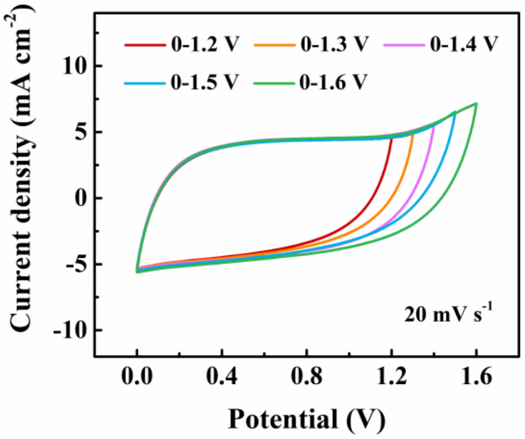

Fig. S17 The CV curves of the PMP DM ICH SC at various voltage windows (the scan rate of 20 mV s^{-1})

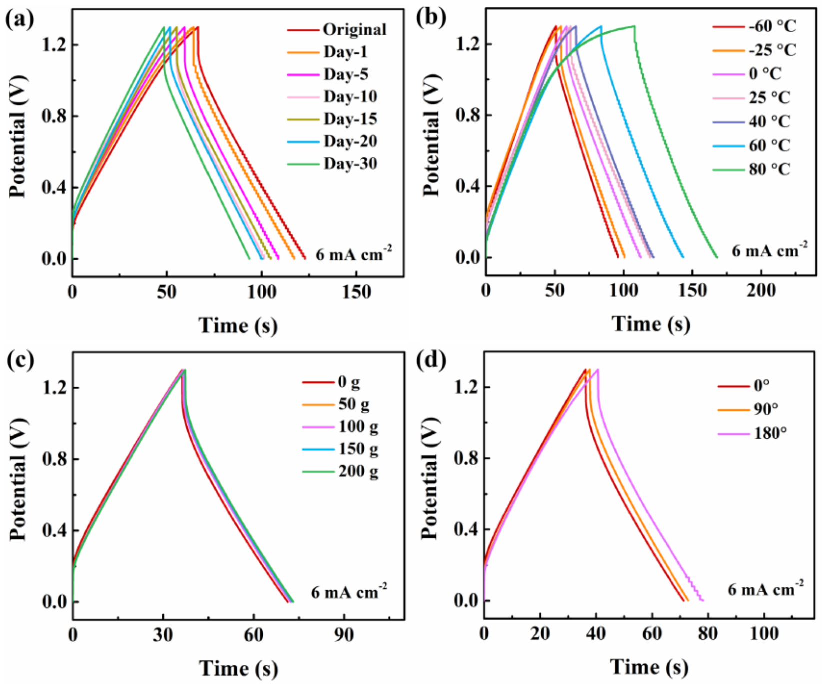

Fig. S18 GCD profile of the PMP DN ICH SC **a** at different stroge days, **b** within various temperatures, **c** under different pressures, **d** at different bending angles

Table S1 Comparison of the temperature coefficient of resistance (TCR) of the PMP DN ICH with other reported previously representative hydrogel materials

Electrolyte	Temperature range (ºC)	TCR (%/ºC)	Refs.
PMP DN ICH	30–60	−1.96	This work
PAA–Zr^{4+}/Gly/IL gel	0–50	−1.891	[S16]
PDA–rGO/SA/PAM organohydrogel–1	15–60	−1.45	[S17]
PS/PPy/CNTs hydrogel	14.5–68.2	−0.56	[S18]
TG2P3 hydrogel	20–80	−1.2	[S19]
PVA/PAS–PPy gel	25–75	−0.64	[S20]
Ionohydrogels ($Al_{2.8}IL_{25}$)	0–45	−0.035	[S21]
PCOH–8	15–60	−1.64	[S22]
CH–GT hydrogel	20–100	−0.83	[S23]
TA@HAP NWs–PVA(W/EG) hydrogel	30–80	−0.536	[S24]
PNA/PVP/TA/Fe^{3+} 3:5 hydrogel	30–37	−1.39	[S25]

Table S2 Comparison of the electrochemical performance of the PMP DN ICH SC with other representative hydrogel materials-based SC reported previously

Electrolyte	Current density	Capacitance	Potential window (V)	Temperature range (°C)	Refs.
PMP DN ICH	1 mA cm^{-2}	263.08 mF cm^{-2}	0–1.3	−60–80	This work
	20 mV s^{-1}	57.00 mF cm^{-2}			
PAA–PVA/PAM/Zn^{2+} organohydrogel	2 A g^{-1}	40 mAh g^{-1}	0–1.8	−25–25	[S26]
PVA/Agar–EMIMBF$_4$–Li_2SO_4	0.3 A g^{-1}	28.8 F g^{-1}	0–1.0	−30–80	[S27]
PEI–PVA–Bn–LiCl	20 mV s^{-1}	16.7 mF cm^{-2}	0–1.4	25	[S28]
PMEL	0.5 mA cm^{-2}	243.3 mF cm^{-2}	0–0.8	−10–80	[S29]
PVA/PAMAA/Gly/ NaCl organohydrogel	0.5 mA cm^{-2}	75.75 mF cm^{-2}	0–1.0	−20–25	[S10]
PHAA$_{15}$N$_{40}$/PDA$_{150}$	0.025 A cm^{-1}	0.37 F g^{-1}	0–1.0	0–90	[S30]
DES	0.5 A g^{-1}	71.52 F g^{-1}	0–1.2	−20–80	[S31]
H_2SO_4/PVA	0.5 mA cm^{-2}	299.79 mF cm^{-2}	0–0.8		[S20]
EPY	0.8 mA cm^{-2}	212 mF cm^{-2}	0–0.8	−30–60	[S32]
PANI–PPG	0.2 mA cm^{-2}	95.8 mF cm^{-2}	0–0.8	−60–100	[S33]
XG–ionogel	10 mV s^{-1}	41 mF cm^{-2}	0–2.5	−40–100	[S34]
MMT/PVA	0.05 mA cm^{-2}	2.89 mF cm^{-2}	0–0.8		[S35]

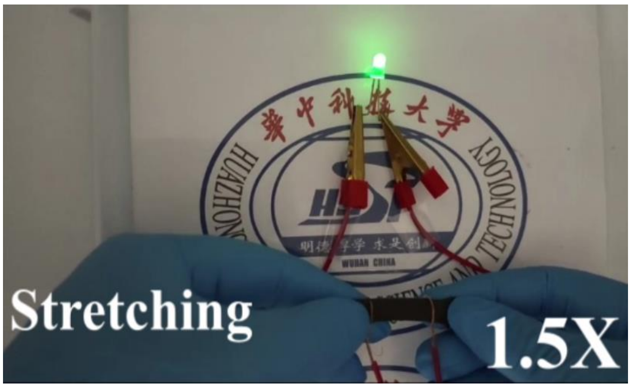

Movie S1 The luminance of LEDs (working voltage of 3.0 V) by using PMP DN ICH as conductor at different tensile states

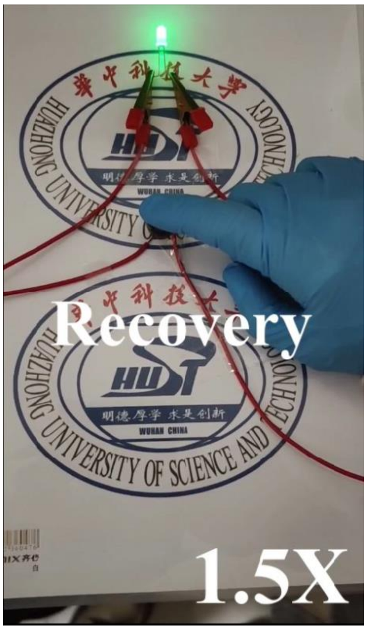

Movie S2 The luminance of LEDs (working voltage of 3.0 V) by using PMP DN ICH as conductor at different compression states

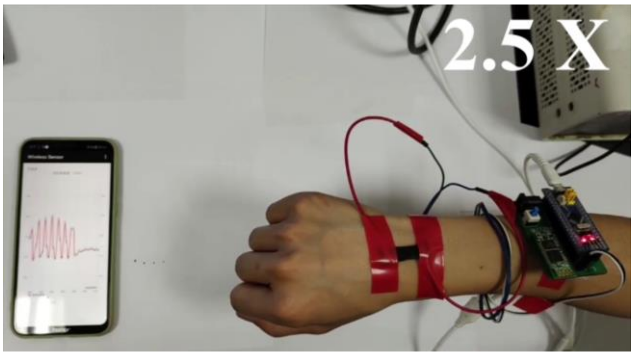

Movie S3 Monitoring of the repeated wrist movements using a wireless wearable sensor

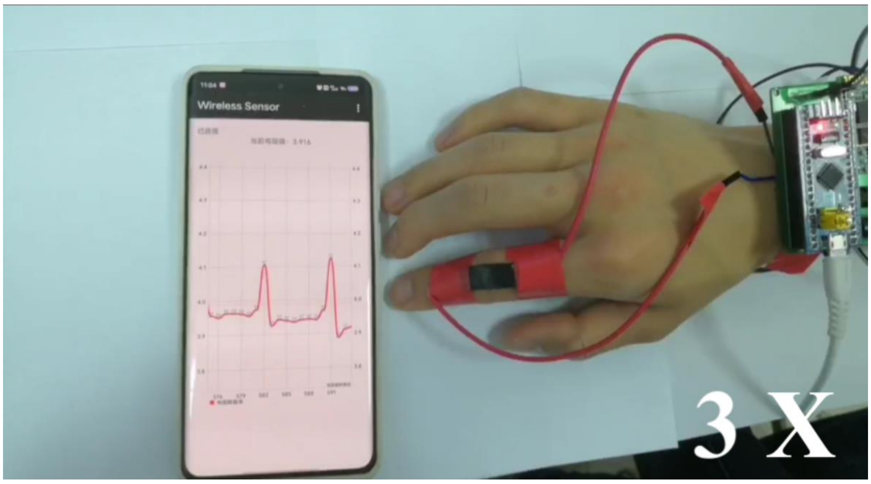

Movie S4 Monitoring of finger bending with different bending angles using a wireless wearable sensor

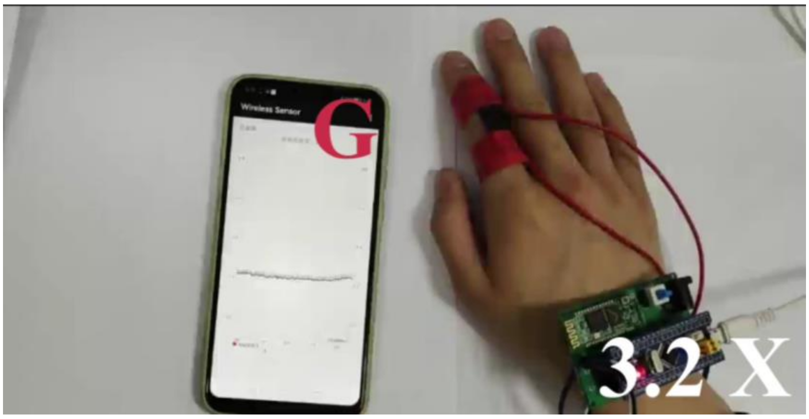

Movie S5 Monitoring of the transmission of distress signals of “GO” using a wireless wearable sensor

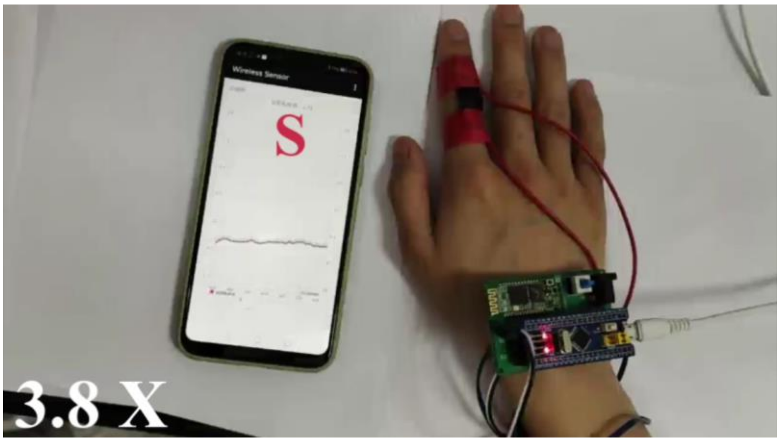

Movie S6 Monitoring of the transmission of distress signals of “SOS” using a wireless wearable sensor

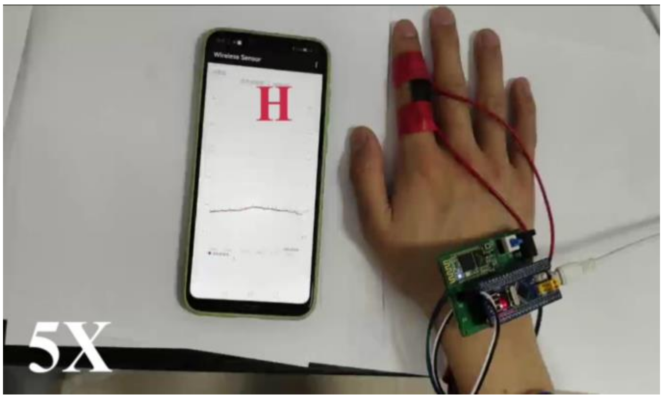

Movie S7 Monitoring of the transmission of distress signals of “HELP” using a wireless wearable sensor

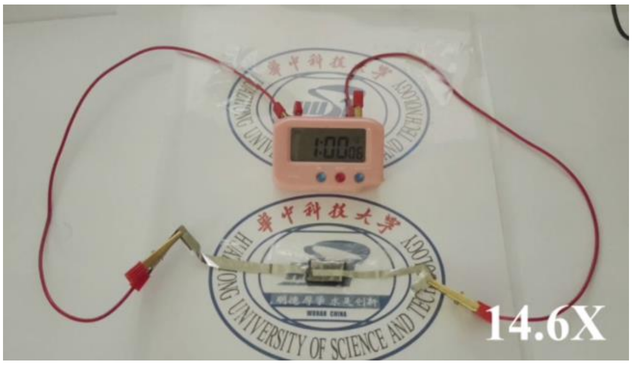

Movie S8 The PMP DM ICH SC powering an electronic meter

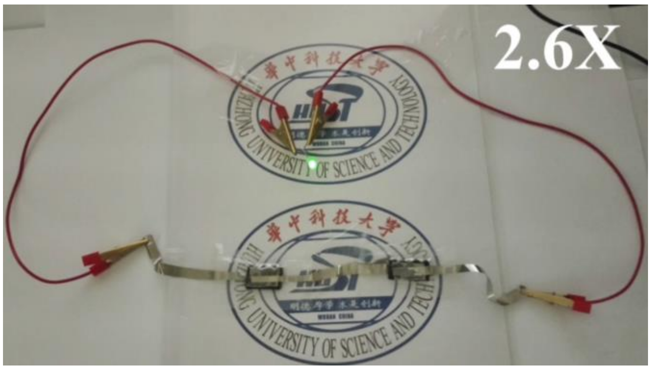

Movie S9 The PMP DM ICH SC powering a LED bulb

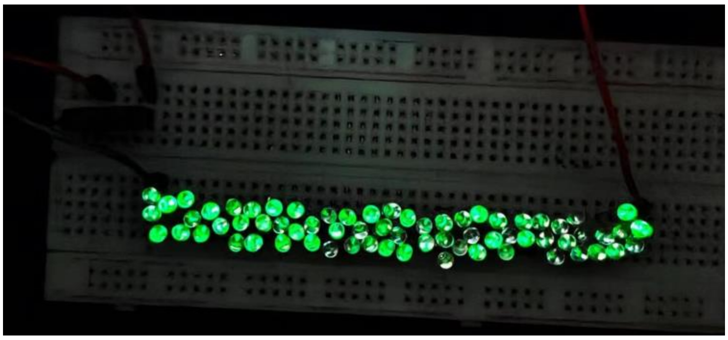

Movie S10 The PMP DM ICH–TENG powering 68 LED bulbs

Supplementary References

[S1] K. Liang, H. Toghiani, G. Li, C.U. Pittman Jr, Synthesis, morphology, and viscoelastic properties of cyanate ester/polyhedral oligomeric silsesquioxane nanocomposites. J. Polym. Sci. A Polym. Chem. **43**, 3887–3898 (2005). https://doi.org/10.1002/pola.20861

[S2] Y. Wang, J. Qiu, J. Peng, J. Li, M. Zhai, One-step radiation synthesis of gel polymer electrolytes with high ionic conductivity for lithium-ion batteries. J. Mater. Chem. A **5**, 12393–12399 (2017). https://doi.org/10.1039/C7TA02291C

[S3] X. Luo, L. Zhu, Y.-C. Wang, J. Li, J. Nie et al., A flexible multifunctional triboelectric nanogenerator based on MXene/PVA hydrogel. Adv. Funct. Mater. **31**, 2104928 (2021). https://doi.org/10.1002/adfm.202104928

[S4] Y. Li, J. Yan, Y. Liu, X.-M. Xie, Super tough and intelligent multibond network physical hydrogels facilitated by $Ti_3C_2T_x$ MXene nanosheets. ACS Nano **16**, 1567–1577 (2022). https://doi.org/10.1021/acsnano.1c10151

[S5] W. Yuan, X. Qu, Y. Lu, W. Zhao, Y. Ren et al., MXene-composited highly stretchable, sensitive and durable hydrogel for flexible strain sensors. Chin. Chem. Lett. **32**, 2021–2026 (2021). https://doi.org/10.1016/j.cclet.2020.12.003

[S6] W. Zhao, J. Jiang, W. Chen, Y. He, T. Lin et al., Radiation synthesis of rapidly self-healing, durable, and flexible poly(ionic liquid)/MXene gels with anti-freezing property for multi-functional strain sensors. Chem. Eng. J. **468**, 143660 (2023). https://doi.org/10.1016/j.cej.2023.143660

[S7] Y. Ye, Y. Zhang, Y. Chen, X. Han, F. Jiang, Cellulose nanofibrils enhanced, strong, stretchable, freezing-tolerant ionic conductive organohydrogel for multi-functional sensors. Adv. Funct. Mater. **30**, 2003430 (2020). https://doi.org/10.1002/adfm.202003430

[S8] Y. Sun, Y. Wang, Y. Liu, S. Wu, S. Zhang et al., Biomimetic chromotropic photonic-ionic skin with robust resilience, adhesion, and stability. Adv. Funct. Mater. **32**, 2204467 (2022). https://doi.org/10.1002/adfm.202204467

[S9] H. Zhou, J. Lai, B. Zheng, X. Jin, G. Zhao et al., From glutinous-rice-inspired adhesive organohydrogels to flexible electronic devices toward wearable sensing, power supply, and energy storage. Adv. Funct. Mater. **32**, 2108423 (2022). https://doi.org/10.1002/adfm.202108423

[S10] J. Huang, S. Peng, J. Gu, G. Chen, J. Gao et al., Self-powered integrated system of a strain sensor and flexible all-solid-state supercapacitor by using a high performance ionic organohydrogel. Mater. Horiz. **7**, 2085–2096 (2020). https://doi.org/10.1039/D0MH00100G

[S11] X. Li, L. Kong, G. Gao, A bio-inspired self-recoverable polyampholyte hydrogel with low temperature sensing. J. Mater. Chem. B **9**, 2010–2015 (2021).

https://doi.org/10.1039/D0TB02895A

[S12] J. Yu, Y. Feng, D. Sun, W. Ren, C. Shao et al., Highly conductive and mechanically robust cellulose nanocomposite hydrogels with antifreezing and antidehydration performances for flexible humidity sensors. ACS Appl. Mater. Interfaces **14**, 10886–10897 (2022). https://doi.org/10.1021/acsami.2c00513

[S13] Y. Liu, W. Wang, K. Gu, J. Yao, Z. Shao et al., Poly(vinyl alcohol) hydrogels with integrated toughness, conductivity, and freezing tolerance based on ionic liquid/water binary solvent systems. ACS Appl. Mater. Interfaces **13**, 29008–29020 (2021). https://doi.org/10.1021/acsami.1c09006

[S14] Y. Feng, J. Yu, D. Sun, W. Ren, C. Shao et al., Solvent-induced *in situ* self-assembly lignin nanoparticles to reinforce conductive nanocomposite organogels as anti-freezing and anti-dehydration flexible strain sensors. Chem. Eng. J. **433**, 133202 (2022). https://doi.org/10.1016/j.cej.2021.133202

[S15] X. Yao, S. Zhang, L. Qian, N. Wei, V. Nica et al., Super stretchable, self-healing, adhesive ionic conductive hydrogels based on tailor-made ionic liquid for high-performance strain sensors. Adv. Funct. Mater. **32**, 2204565 (2022). https://doi.org/10.1002/adfm.202204565

[S16] E. Feng, X. Li, X. Li, M. Zhang, L. Cao et al., Toughened, self-healing and self-adhesive conductive gels with extraordinary temperature adaptability for dual-responsive sensors. J. Mater. Chem. A **10**, 25527–25538 (2022). https://doi.org/10.1039/D2TA07961E

[S17] Z. Xie, H. Li, H.-Y. Mi, P.-Y. Feng, Y. Liu et al., Freezing-tolerant, widely detectable and ultra-sensitive composite organohydrogel for multiple sensing applications. J. Mater. Chem. C **9**, 10127–10137 (2021). https://doi.org/10.1039/D1TC02599F

[S18] A. Abodurexiti, X. Maimaitiyiming, Carbon nanotubes-based 3D printing ink for multifunctional “artificial epidermis” with long-term environmental stability. Macromol. Chem. Phys. **223**(11), 2100486 (2022). https://doi.org/10.1002/macp.202100486

[S19] X. Huang, G. Ge, M. She, Q. Ma, Y. Lu et al., Self-healing hydrogel with multiple dynamic interactions for multifunctional epidermal sensor. Appl. Surf. Sci. **598**, 153803 (2022). https://doi.org/10.1016/j.apsusc.2022.153803

[S20] S. Wang, D. Zhang, X. He, J. Zhou, Y. Zhou et al., Anti-swelling zwitterionic hydrogels as multi-modal underwater sensors and all-in-one supercapacitors. ACS Appl. Polym. Mater. **4**, 7498–7507 (2022). https://doi.org/10.1021/acsapm.2c01202

[S21] X. Zhang, C. Cui, S. Chen, L. Meng, H. Zhao et al., Adhesive ionohydrogels based on ionic liquid/water binary solvents with freezing tolerance for flexible ionotronic devices. Chem. Mater. **34**, 1065–1077 (2022).

https://doi.org/10.1021/acs.chemmater.1c03386

[S22] Z. Xie, Z. Chen, X. Hu, H.-Y. Mi, J. Zou et al., Ultrastretchable, self-healable and adhesive composite organohydrogels with a fast response for human–machine interface applications. J. Mater. Chem. C **10**, 8266–8277 (2022). https://doi.org/10.1039/D2TC00932C

[S23] J. Liu, H. Wang, T. Liu, Q. Wu, Y. Ding et al., Multimodal hydrogel-based respiratory monitoring system for diagnosing obstructive sleep apnea syndrome. Adv. Funct. Mater. **32**, 2204686 (2022). https://doi.org/10.1002/adfm.202204686

[S24] J. Wen, J. Tang, H. Ning, N. Hu, Y. Zhu et al., Multifunctional ionic skin with sensing, UV-filtering, water-retaining, and anti-freezing capabilities. Adv. Funct. Mater. **31**, 2011176 (2021). https://doi.org/10.1002/adfm.202011176

[S25] Q. Pang, H. Hu, H. Zhang, B. Qiao, L. Ma, Temperature-responsive ionic conductive hydrogel for strain and temperature sensors. ACS Appl. Mater. Interfaces **14**(23), 26536–26547 (2022). https://doi.org/10.1021/acsami.2c06952

[S26] B. Zheng, H. Zhou, Z. Wang, Y. Gao, G. Zhao et al., Fishing net-inspired mutiscale ionic organohydrogels with outstanding mechanical robustness for flexible electronic devices. Adv. Funct. Mater. **33**(28), 2213501 (2023). https://doi.org/10.1002/adfm.202213501

[S27] H. Peng, X. Gao, K. Sun, X. Xie, G. Ma et al., Physically cross-linked dual-network hydrogel electrolyte with high self-healing behavior and mechanical strength for wide-temperature tolerant flexible supercapacitor. Chem. Eng. J. **422**, 130353 (2021). https://doi.org/10.1016/j.cej.2021.130353

[S28] J. Liu, J. Huang, Q. Cai, Y. Yang, W. Luo et al., Design of slidable polymer networks: a rational strategy to stretchable, rapid self-healing hydrogel electrolytes for flexible supercapacitors. ACS Appl. Mater. Interfaces **12**, 20479–20489 (2020). https://doi.org/10.1021/acsami.0c03224

[S29] Q. Hu, X. Shi, K. Sun, S. Cui, H.A. Hamouda et al., A super-stretchable and thermally stable hydrogel electrolyte for high performance supercapacitor with wide operation temperature. J. Alloys Compd. **909**, 164646 (2022). https://doi.org/10.1016/j.jallcom.2022.164646

[S30] Z. Gao, L. Kong, R. Jin, X. Liu, W. Hu et al., Mechanical, adhesive and self-healing ionic liquid hydrogels for electrolytes and flexible strain sensors. J. Mater. Chem. C **8**, 11119–11127 (2020). https://doi.org/10.1039/D0TC01094D

[S31] X. Bu, Y. Ge, L. Wang, L. Wu, X. Ma et al., Design of highly stretchable deep eutectic solvent-based ionic gel electrolyte with high ionic conductivity by the addition of zwitterion ion dissociators for flexible supercapacitor. Polym. Eng. Sci. **61**, 154–166 (2021). https://doi.org/10.1002/pen.25564

[S32] Q. Hu, S. Cui, K. Sun, X. Shi, M. Zhang et al., An antifreezing and thermally

stable hydrogel electrolyte for high-performance all-in-one flexible supercapacitor. J. Energy Storage **50**, 104231 (2022). https://doi.org/10.1016/j.est.2022.104231

[S33] J. Huang, S. Han, J. Zhu, Q. Wu, H. Chen et al., Mechanically stable all flexible supercapacitors with fracture and fatigue resistance under harsh temperatures (adv. funct. mater. 35/2022). Adv. Funct. Mater. **32**, 2270200 (2022). https://doi.org/10.1002/adfm.202270200

[S34] C. Yin, X. Liu, J. Wei, R. Tan, J. Zhou et al., "All-in-Gel" design for supercapacitors towards solid-state energy devices with thermal and mechanical compliance. J. Mater. Chem. A **7**, 8826–8831 (2019). https://doi.org/10.1039/C9TA01155B

[S35] S. Wu, L. Tang, Y. Xu, J. Yao, G. Tang et al., A self-powered flexible sensing system based on a super-tough, high ionic conductivity supercapacitor and a rapid self-recovering fully physically crosslinked double network hydrogel. J. Mater. Chem. C **10**, 3027–3035 (2022). https://doi.org/10.1039/D1TC04514H
